# Committing to the wrong artificial delegate in a collective-risk dilemma is better than directly committing mistakes

**DOI:** 10.1038/s41598-024-61153-9

**Published:** 2024-05-07

**Authors:** Inês Terrucha, Elias Fernández Domingos, Pieter Simoens, Tom Lenaerts

**Affiliations:** 1 Department of Information Technology-IDLab, Ghent University-IMEC, Technologiepark Zwijnaarde 126, 9052 Ghent, Belgium; 2 Artificial Intelligence Lab, Computer Science Department, Vrije Universiteit Brussel, 1050 Brussels, Belgium; 3 Machine Learning Group, Département d’Informatique, Université Libre de Bruxelles, 1050 Brussels, Belgium; 4 FARI Institute, Université Libre de Bruxelles-Vrije Universiteit Brussel, 1050 Brussels, Belgium; 5 Center for Human-Compatible AI, UC Berkeley, Berkeley, 94702 USA

**Keywords:** Evolutionary theory, Human behaviour, Social evolution

## Abstract

While autonomous artificial agents are assumed to perfectly execute the strategies they are programmed with, humans who design them may make mistakes. These mistakes may lead to a misalignment between the humans’ intended goals and their agents’ observed behavior, a problem of value alignment. Such an alignment problem may have particularly strong consequences when these autonomous systems are used in social contexts that involve some form of collective risk. By means of an evolutionary game theoretical model, we investigate whether errors in the configuration of artificial agents change the outcome of a collective-risk dilemma, in comparison to a scenario with no delegation. Delegation is here distinguished from no-delegation simply by the moment at which a mistake occurs: either when programming/choosing the agent (in case of delegation) or when executing the actions at each round of the game (in case of no-delegation). We find that, while errors decrease success rate, it is better to delegate and commit to a somewhat flawed strategy, perfectly executed by an autonomous agent, than to commit execution errors directly. Our model also shows that in the long-term, delegation strategies should be favored over no-delegation, if given the choice.

## Introduction

Recent advances made within the context of automated decision-making and artificial intelligence (AI) have shifted commonly held beliefs about certain tasks requiring human intelligence and creativity. For example, the public deployment of large language models that write code and text^1–3^ have showcased that AI has the capacity to automate even tasks that were thought to require some degree of imagination. The excitement surrounding these new technologies has led to the belief that autonomous systems embedded with AI may take over a plethora of different tasks, even within the context of social and moral decision-making. Additionally, the transition towards a human-to-AI delegation future may even be accelerated in situations where risk is involved, as Liehner et al.^4^ have shown that in cases of high risk of material loss, delegation to artificial agents is more likely.

Nonetheless, individuals may commit errors when they delegate, even to autonomous agents^5–8^. A person may, for example, select an artificial delegate that does not match their goals by not fully understanding what the agent will do, or may fail to correctly configure their behavior due the complexity of the parameters or of the user interface, or because they lack in the understanding how each parameter affects the outcome. As a consequence, the agents chosen or configured by humans may not be aligned with their principal’s intended preferences. This communication problem, well-known in human-to-human delegation^9^, corresponds to the problem of AI value alignment in the context of human-to-AI delegation^10,11^, an issue actively studied within the human-AI interaction research community^12–14^. Understanding how choices in delegation affect the alignment in terms of outcome is thus fundamental.

**Figure 1:**
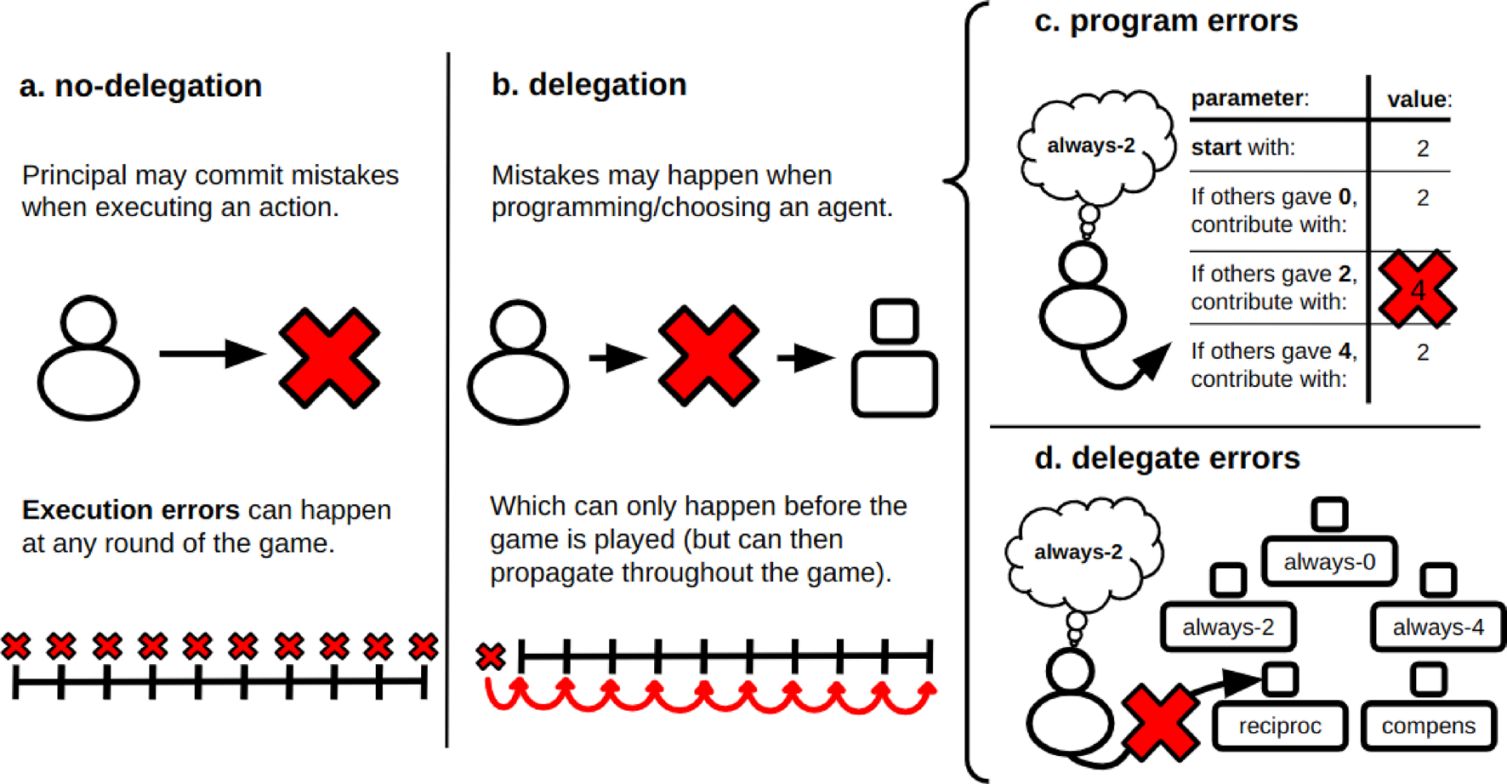
No-delegation vs delegation when playing a strategic game. Distinguishing between (**a**) no-delegation and (**b**) delegation is done here through the timing at which errors may occur. For the case of no-delegation, the illustration in (**a**) shows an individual directly committing a mistake and the timing illustration of that mistake with regards to the game being played, specifically it is shown that the mistake may occur at any round of a multiple-round game. In (**b**), delegation is represented by a scheme of an individual committing mistakes during the process of delegating to an agent and the timing representation of that moment happening before the multiple-rounds of the game are played. Nonetheless, it is important to note that an error committed when delegating to an agent might lead to the propagation of that error throughout the game as the wrong agent might result (or not) in a completely different course of action than the one originally intended by the human principal. Moreover, it is shown how a programming error (**c**) and a delegate error (**d**) would occur. Specifically, in (**c**) it can be observed that a programming error may occur when an individual is “filling” each parameter that defines their agent’s simple program, by mistakenly choosing a value that does not correspond to their intended strategy. Instead, (**d**) shows that a delegate error occurs when an individual wrongly chooses an agent already programmed that does not correspond to their intended strategy. In both cases (**c**) and (**d**), the intended strategies are represented by a thought balloon.

In game theory there is so far no formal way to distinguish between the direct action (no-delegation) of a principal playing a game and a strategy programmed by them to play the game on their behalf (delegation)^15,16^. Yet, empirical differences are often observed in behavioral experiments that either use the direct or the strategy method^15–19^ (which we interpret respectively as no-delegation vs delegation, since no significant difference was found between the strategy method and delegation to autonomous agents in ref.^18^). We provide a game theoretical model that differentiates between no-delegation and delegation through when mistakes occur in the decision-making process: when players do not delegate, mistakes may occur during the game (Fig. 1a), while when they delegate by selecting (or configuring) an agent with a strategy, mistakes can only occur before the game (Fig. 1b). In the latter case, the actual error rate might be different than the probability of a mistake occurring, as a strategy different than the one actually intended by the principal will then be implemented, which might result in more (or less) mistakes during the game. Of course one can also consider differences based on the control the principal has over the behavior of the delegate: as also shown in Fig. 1c,d, a mistake can be made in the selection of a pre-programmed delegate, or in how the behavior of the delegate is programmed parameter-by-parameter. This work will investigate how errors in no-delegation affect outcomes differently from errors in these two forms of delegation in strategic interactions.

We adopt an evolutionary game-theoretical (EGT) approach^20–22^, building on prior literature in the same context^23–25^. We aim to improve the understanding of the AI value alignment problem by systematically studying if the errors made by autonomous systems will hinder (or enhance) outcomes beneficial for society. Additionally, we evaluate whether, in the long term, to delegate will become the dominant choice. An EGT approach provides a big picture perspective of how behavior may spread in a society where individuals may adopt the behavior of their more successful peers. Different from other adaptive approaches, EGT considers the population-level effects, studying how frequencies of strategies change over time, providing thus insight into the likelihood of adopting diverging strategies (e.g. delegation vs. no-delegation) in a society.

To ground the delegation framework presented here, a use case is made out of the collective-risk dilemma (CRD)^19,26,27^. The reason for this choice is that the alignment problem may have particularly strong consequences when autonomous systems are employed in social contexts that involve the realization of a shared goal which incorporates a notion of collective risk, as was also argued in Liehner et al.^4^. In the CRD (see Fig. S1 for an illustration of this game) a group of participants are given an initial private endowment, and are then confronted over a finite number of rounds with the decision of how much to contribute (in our case, a choice between actions 0, 2 and 4 but any other set would work, see "Methods") to a public good. This is reminiscent of goal-oriented tasks assigned to AI systems, that have a limited amount of time to achieve a result. If the group is able to meet a known threshold by the end of the last round, the group is considered successful and each participant shares in the benefits. If on the contrary, they fail to meet this threshold, there is a certain risk probability that everyone will lose the remainder of their endowments (see CRD subsection in "Methods" for more details on how to formalize the game’s payoffs).

The choice of using the CRD specifically for the present delegation study is thus motivated by a series of arguments: (i) it is a game consisting of multiple rounds, where therefore it makes sense that a principal would want to automate their decision process (in order to engage in other parallel activities or simply to release themselves from such decision fatigue); (ii) the outcome of the game can only be evaluated in the end. Interrupting the course of action of a delegate during the game would make little sense as one can only grasp the outcome when the game ends. As such, the game reflect a human-outside-of-the-loop scenario; (iii) it has a risky outcome, making it relevant for delegation research, as expressed in Liehner et al.^4^ and (iv) there is moreover experimental data available^19^, allowing thus for the comparison of the model’s results with data.

Following the EGT methodology (more details in subsection “Evolutionary game theory and small mutation limit” in Methods), we define a set of strategies that are expected to compete within an evolving population playing the CRD. Although a multitude of behavioral profiles can emerge when considering a multi-round game, where many different interests may be at conflict, we follow previous CRD research^19,27,28^ and restrict the current study to a set of 5 behavioral profiles (and see how we implement them in Fig. S2 in the SI): Reciprocal, Compensatory, (fixed-)selfish (or always-0), (fixed-)fair (or always-2) and (fixed-)altruistic behavior (or always-4). Where the first two behaviors are conditional on the previous choices in the group, the latter three consider (fixed) unconditional behavior. In this manner, the insights provided by the theoretical model are more easily comparable to the experimental results already reported^19^ on delegation. In Figs. S2 and S3 we show a representation of the above mentioned 5 simple programs and how they would work when playing the CRD (see also subsection “Strategy definition and agent’s settings” in Methods for more detail).

On top of the behavioral profiles defined above, the model introduces three error types/modes in order to differentiate between situations corresponding to no delegation and delegation. Specifically, we consider 3 types of errors: *execution errors* in the case of no-delegation mimicking the idea that at each round a person can make a mistake, *program errors* when one programs an agent to which to delegate, and *delegate errors* when one chooses the wrong agent from a pool of pre-programmed agents where all 5 behaviors are represented (see panels c and d in Fig. 1 for a representation of the programming and the delegate errors).

In order to better understand how these different errors are implemented in our model, consider for example an individual whose preferred strategy is “always-2”. An execution (no-delegation) error, in this case, would consist in the probability of this individual erroneously contributing 4 (or 0) in one (or more) rounds of the game, even though their strategy prescribes a 2 at every round. In delegation, if a user is required to configure the behavior of their agent by specifying what to do based on observations about the other agents, a program error would consist in the principal setting wrongly one of their agent’s parameters (see Fig. 1c where an individual who intends to play with “always-2” actually sets the third parameter to 4 instead of 2). Finally, if delegation happens by requiring the principal to just select a predefined agent, a delegate error would consist in the principal wrongly selecting a “reciprocal” agent (for example), instead of their intended “always-2” agent (see Fig. 1d for an illustration of this type of error). It may happen that an error in configuring the agent will not lead to changes in the actions when compared with the originally intended strategy. Other times, such an error might be detrimental and instead deviate all actions from what the principal would have intended. In general, these errors can be seen as similar to the trembling hand effect well studied in game theory^6,7,29–31^.

Note that when no errors are committed, all strategies are in fact indistinguishable, and they are represented by memory-1 strategies consisting of simple programs that can be defined by the parameters: how much to contribute in round 0, and how much to contribute if others have contributed 0, 2 or 4 on the previous round (see Fig. S2 in the SI where all the 5 behavioral profiles are represented by their respective simple programs). Since in the case where no errors are committed the ambiguity between the no-delegation and the two delegation scenarios is recovered, any difference between the outcomes of the evolution of the three different population scenarios (one for each different error mode) can only be attributed to the type of error that was made, and thus also to whether delegation was used or not. To facilitate this comparison, we compare the three populations on CRD game related metrics such as group success in avoiding collective disaster, or total contributions to the public account by the end of the game (see Methods for a more detailed explanation of the metrics).

Finally, to analyze the viability of delegation to autonomous agents to social contexts in the long-term, the last section of results is dedicated to analyzing the case of optional delegation. To tackle this research question, the error mode is added to the strategy of each individual within the evolving population. Considering delegation as part of the competing strategies allows one to determine the delegation rate (see Eq. (4) for more detail) that a population is expected to eventually reach, providing insight into when delegation is more favorable and thus likely to be chosen. After all, it is not sufficient to understand how delegation shapes decision-making in mixed motive scenarios, it is also essential to answer whether or not it will even be adopted in this context.

In this manner, the main contributions of this work read as follows:

- We introduce a novel model that allows us to distinguish between delegation and no-delegation within a game-theoretical framework;
- By applying this model to the CRD game, we find that for a wide range of error probabilities, delegation leads to higher success rates when mistakes are possible;
- Public account values do not follow the same trends as success rates, showing the importance of always analyzing both trends separately;
- When considering delegation as optional rather than mandatory within a population, we show that introducing pre-set agents would lead to more people delegating than if they have to program their own agents.

## Results and discussion

### Delegation is more successful when mistakes are possible

Are populations that delegate more successful (in avoiding a collective risk) than those who do not? Are groups in these populations more pro-social in the sense that they contribute more to the public good? To answer these questions, the success rate (i.e. the likelihood of reaching the threshold in the CRD) and the average public account contributions (see Methods for details) are compared between three evolving populations: one where *execution errors* are committed (in this case there is no delegation); one where individuals program agents to make their contributions and can therefore commit *program errors* (a case of delegation); and one where individuals simply choose from a pool of pre-set agents (another case of delegation) and are thus liable to choosing the wrong agent by mistake in what was denominated *delegate errors*.

As also explained in the introduction, in all of these populations there are the same 5 competing behavioral profiles, which are assumed to be the individuals’ true preferences when playing the CRD (and supported by experimental data^19^): **R**eciprocal (players start by contributing 2 and then proceed to contribute 0, 2 or 4 if others contributed in the previous round 0, 2 or 4, respectively), **C**ompensatory (players start by contributing 2 and then proceed to contribute 4, 2 or 0 if others contributed in the previous round 0, 2 or 4, respectively), always-**0** (players always contribute 0), always-**2** (players always contribute 2) and always-**4** (players always contribute 4). As explained in Methods, using the stationary distribution of these strategies (denoted by $\sigma _i$ for each strategy *i*) resulting from the evolutionary process that each population goes through, the success rate (Eq. 2) and the average public account contributions (Eq. 3) associated with each mode of delegation that we consider in this manuscript can be calculated.

The fitness of the competing strategies within the population is calculated as the expected payoff obtained by each strategy while engaging in a CRD played between 6 players, who all start with an initial endowment of 40 and must collectively reach a value of 120 in the public account after 10 rounds of play. In each round of play, the participants may only contribute 0, 2 or 4 similarly to what has been done in experimental work^19,26,27^ to facilitate comparison with the model results. If the group fails to meet this threshold, there is a risk probability $p=0.9$ that everyone will lose the remainder of their endowments, which places this analysis in the realm of high-risk scenarios (a case widely studied in behavioral experiments due to the high stakes that it intends to abstract^19,26,27^). In the SI it is shown how the high-risk scenario is the most interesting case to study delegation vs. no-delegation in the context of the CRD (Figs. S8–15 in the SI).

**Figure 2:**
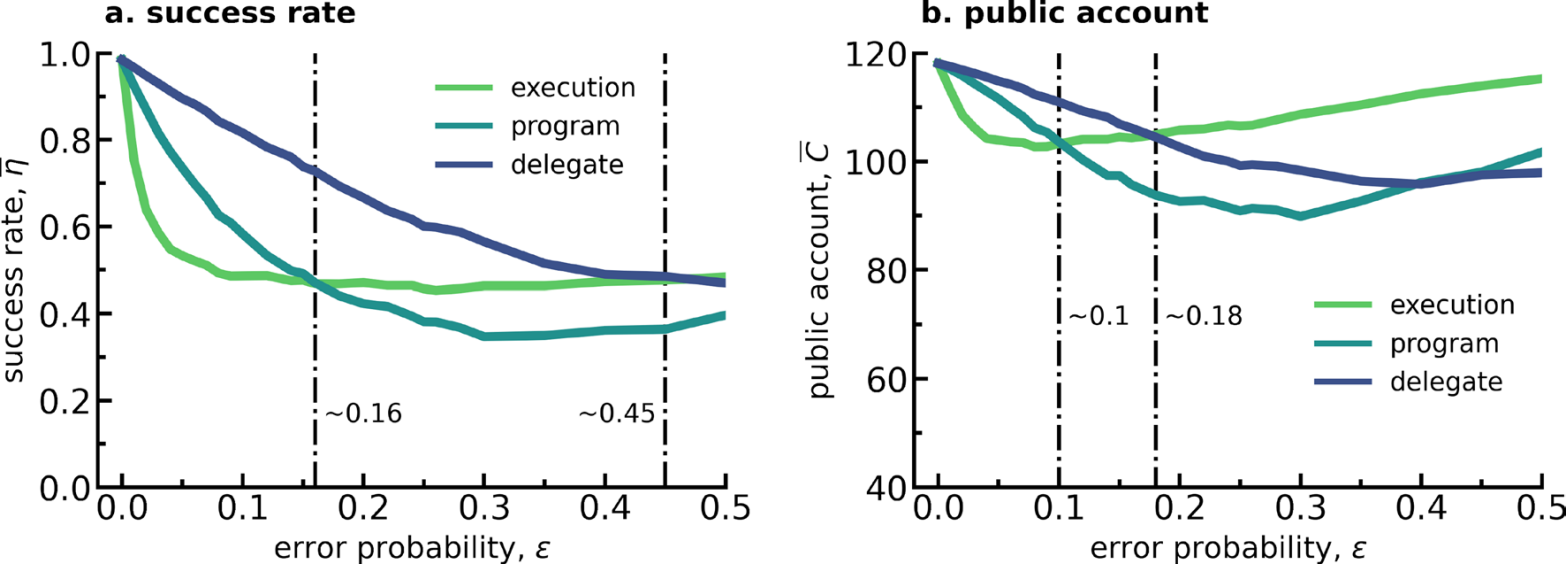
Success rate and average public account values observed in 3 populations distinguished by the type of errors their individuals commit. Following the color legend inside each sub-figure, the errors can be of type *execution*, *program* and *delegate*. Sub-figure (**a**) refers to success rate in terms of error probability $\epsilon$. In (**a**), two dotted-dashed lines at $x\sim 0.16$ and $x\sim 0.45$ indicate the value of $\epsilon$ until which the program and the delegate population (both representing a type of delegation), respectively, achieve higher success rates than the execution (no-delegation) population. Sub-figure (**b**) refers to (average) public account in terms of error probability $\epsilon$. Again in (**b**), with two dotted-dashed lines we indicate the value of $\epsilon$ until which the program ($\epsilon \sim 0.1$) and the delegate population ($\epsilon \sim 0.18$) constitute more pro-social groups (that on average contribute more to the public account) than the execution population. To produce this figure, the following parameters were used: $\beta =0.05$, $Z=100$, $\#sim=10000$, $p=0.9$, $r=10$, $E=40$, $A={0, 2, 4}$, $N=6$.

In Fig. 2 one can observe the success rate and average public account contributions, respectively in panels a. and b., in relation to the error probability for each of the 3 delegation modes. This figure, as well as the others in the Result section, focuses on error probabilities $\epsilon$ within the range $0\le \epsilon \le 0.5$ (the results for the whole range provided in the Supplemental Information (SI), see Figs. S4–7). There are two reasons for this focus: first, an error probability exceeding 0.5 may be considered intentional rather than by chance, which is out of scope for this work. Second, Figs. S8–13 corroborate that for higher error values it becomes unlikely to draw relevant insights, as behavioral differences are no longer driving the dynamics.

Focusing firstly on Fig. 2a, the average success rate $\overline{\eta }$ is shown to be highest when error probability $\epsilon =0$, so when individuals make no mistakes, and thus perform optimally to achieve the goal of the CRD. The figure shows moreover that the introduction of any type of error will result in a decrease of success rate in comparison to what is expected in case of perfect strategy execution. In this way, the existence of errors in the execution of participants’ strategies when playing the CRD in behavioral experiments^19,26,27^ might be the reason that the participants do not achieve success rates as high as the ones predicted by theoretical models that do not include the presence of such errors^19,28^.

Moreover, the success rate of the *execution errors* population (thus no-delegation) drops more rapidly than any of the two of delegation results for $\epsilon < 0.16$. Specifically, the *program errors* population appears to produce more successful outcomes than the *execution errors* population for error probabilities up to $\epsilon \approx 0.16$, whereas the *delegate errors* population achieves higher success rates than either of the other two for error probabilities up to $\epsilon \approx 0.45$. These values of $\epsilon$ represent therefore the intersection points between the curves of no-delegation and the two different delegation mechanisms (for *program errors* at $\epsilon \approx 0.16$ and for *delegate errors* at $\epsilon \approx 0.45$), with their position revealing up until where delegation provides better results than no-delegation. Furthermore, their relative position - the fact that the intersection between *program errors* and *execution errors* occurs at a lower $\epsilon$ than the one between *delegate errors* and *execution errors* - shows that when comparing delegation mechanisms, choosing a pre-set agent results in higher success rates than tweaking your own agent for a wide set of error probabilities, at least in CRD like situations.

In the SI complementing this manuscript, it can be observed that these qualitative insights shown in Fig. 2a actually remain robust for different choices of model parameters used, even though the absolute position of the intersection points might change. Specifically, we see in the SI that increasing and decreasing the group size *N* will result in higher and lower intersection values for $\epsilon$ between both the *program* (the *delegate errors*) population and the *execution errors* population (as observed in Figs. S8 and S16 of the SI). Other parameters such as $\beta$ (Figs. S22 and S26 in the SI), *Z* (Figs. S24 and S26 in the SI), *r* (Figs. S9 and S19 in the SI) and $\#actions$ (Figs. S11 and S21 in the SI) do not show such a linear effect - for example the increase of these parameters results in a closer proximity between the two intersection points rather than moving them both towards one direction - but they still conserve qualitatively the results observed in Fig. 2 for the success rates.

### Yet, a higher success rate does not necessarily translate to higher contributions

Turning the analysis to Fig. 2b, where the average group contributions ($\overline{C}$) to the public account is shown in terms of $\epsilon$ for the 3 populations, we see that for $\epsilon \approx 0$ the trends follow similar paths. As soon as the probability of the individuals within the population committing a mistake becomes $>0$, the group contributions expected in the *execution errors* population experience a sharp drop from the case where error probability $\epsilon =0$. Again one can observe that there is always a region of low error probability for which this population returns the lowest average public account values. When comparing to the case of *program errors*, this region extends between 0 and $\sim 0.1$; when comparing with the case of *delegate errors*, between 0 and $\sim 0.18$ as evidenced in the figure through vertical dotted-dashed lines. Unlike the success rate, average public account contributions appear to be more sensitive to changes in game variables such as group size (Figs. S11 and S19 in the SI) and number of rounds (Figs. S12 and S17). Even though there appears to always be some $\epsilon$ space for which similar trends for delegation vs. no-delegation can be found. As detailed in the SI, one can see that when $r=25$ (Fig. S19 in SI) or $N=4$ (Fig. S17 in SI), the region of small $\epsilon$ for which delegation contributes more than no-delegation is so small that it cannot be perceived in the visualization. As shown in Fig. S21 (in the SI), even though the steepness of the curves varies with the granularity in the action space, the variation happens in an equivalent manner in delegation and no-delegation cases so that the intersection of these curves shows no significant change (also see Fig. S13 in the SI). Selection strength $\beta$ and population size *Z* appear to have a similar effect as the number of rounds *r* (see Figs. S23, S25 and S27 in the SI). An increase in all these quantities is associated with the flattening of the delegation/execution error population, so that instead of reproducing the observed trends in Fig. 2b, the point of intersection disappears leaving this population as the highest contributor to the public account for the almost the entire range of error probabilities.

It is interesting to note that the regions where delegation surpasses no-delegation in terms of success rate do not directly translate to higher average contributions. For example, even though the *program errors* population achieves higher success rates than the *execution errors* population for error probabilities $<0.16$, between 0.1 and 0.18, their individuals are expected to make lower group contributions to the public account. Similarly, the *delegate errors* population shows higher success rates until error probabilities reach 0.45, even though their expected group contributions are lower than the ones made by the *execution errors* population from 0.18 onward. Knowing that there exists a theoretical range of error probability $\epsilon$ for which the success rates comparison between delegation and no-delegation do not follow the same trend as the comparison between group contributions, might lead to interesting experiment designs to probe the error probability to which human participants are prone to committing mistakes when playing this type of game.

### Mistakes lead to the emergence of selfish strategies, hindering success in the high-risk CRD

**Figure 3:**
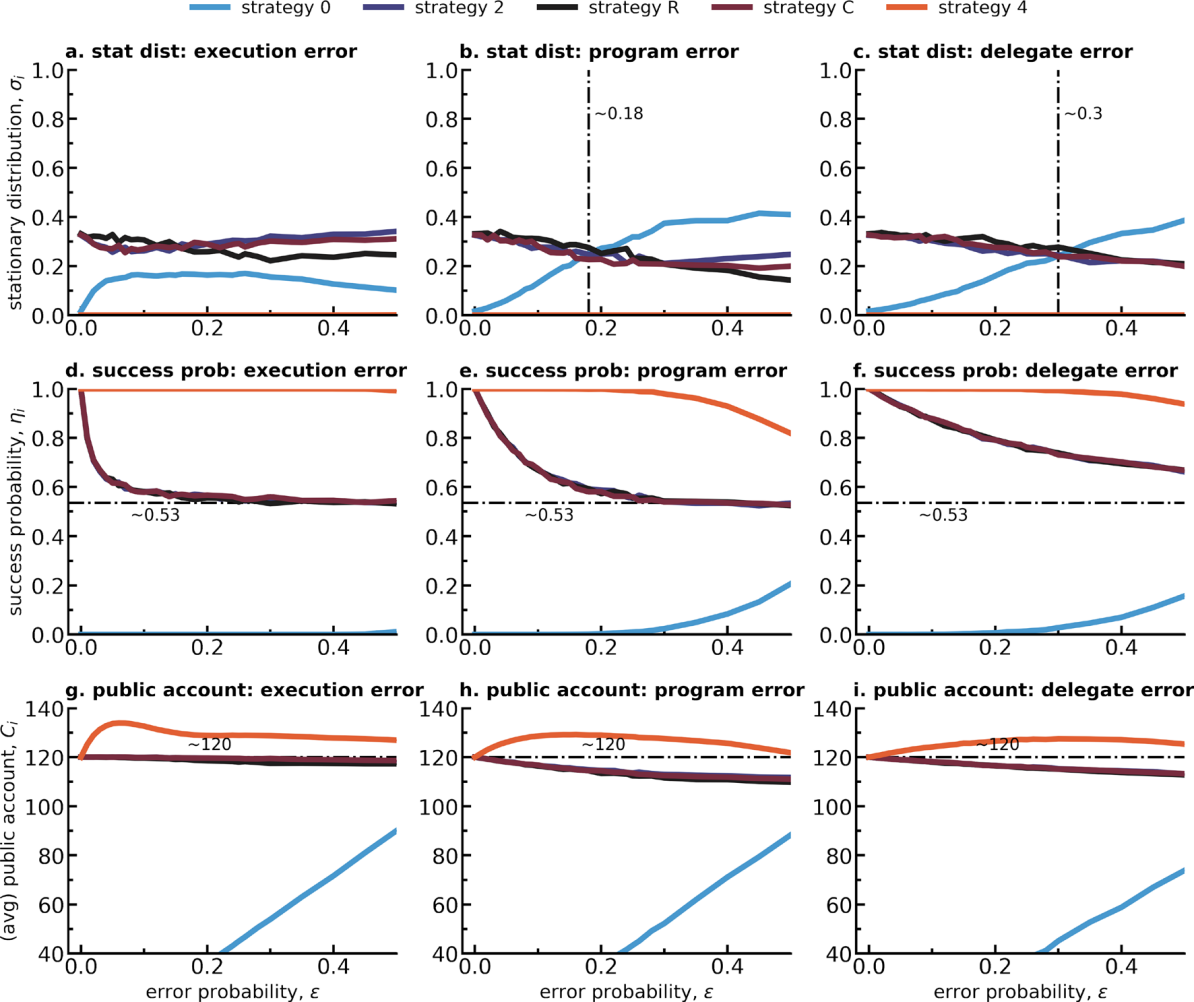
Stationary distribution, success probability and average public account per strategy present in a given error type population. Columns show the different quantities for each error type population: first column execution errors, middle column for program errors and last column for delegate errors. Rows (and y-axis) represent each different quantity of interest: first row shows stationary distribution, second and last show respectively success probability and average public account per each strategy’s monomorphic group as a result of $\#sim=10000$ simulated rounds of play. All quantities are plotted in terms of error probability $\epsilon$. In (**a**) we can observe that strategies *2*, *R* and *C* dominate for every $\epsilon$ even though *0* has a sharp increase immediately when $\epsilon >0$. In (**b**) and (**c**) we denote with a dotted-dashed line at $\epsilon \sim 0.18$ and $\epsilon \sim 0.3$ when strategy *0* becomes the most dominant strategy by surpassing *2*, *R* and *C*. In (**d**–**f**) with an horizontal dotted-dashed line we mark the success probability value observed in populations with execution error (**d**) at $\epsilon =0.5$ to facilitate comparison between sub-figures. Similarly, in (**g**–**i**) we use an horizontal dotted-dashed line to denote the public account threshold (and therefore most efficient group contribution) of $C=120$. This figure was produced with the same parameters as Fig. 2: $\beta =0.05$, $Z=100$, $\#sim=10000$, $p=0.9$, $r=10$, $E=40$, $A={0, 2, 4}$, $N=6$.

In order to better understand how success rate and average public account contributions depend on the error probability $\epsilon$ at the population level, we turn to an analysis of the Eqs. (2) and (3) used to produce the results observed in Fig. 2. These equations show that the population level results depend on the stationary distribution $\sigma _i$ of strategies, the expected group success $\eta _i$ (for the success rate in Fig. 2a) and the average group contributions $C_i$ (for the average public account values in Fig. 2b) of each of the strategies *i* competing within a population, which are: **0** (always-0), **2** (always-2), **4** (always-4), **R** (reciprocal) and **C** (compensatory). In Fig. 3 we show how the stationary distribution of the different strategies vary in function of the error probability $\epsilon$ for each different error type (execution errors in Fig. 3a, program errors in Fig. 3b and delegate errors in Fig. 3c); how success probability and expected public account contributions for each mono-strategic group (i.e. calculated independently of its population evolution) change with $\epsilon$ for all the error types considered (execution in Fig. 3d, program in Fig. 3e and delegate in Fig. 3f for the success probability; execution in Fig. 3g, program in Fig. 3h and delegate in Fig. 3i for the public account values) - see Fig. S5 in the SI to consult the same results within the full range $0\le \epsilon \le 1$. As described in Methods, because a small mutation limit approach^22,32,33^ is used, only monomorphic states of the population are considered, and therefore rely on expected values for mono-strategic groups (groups where only 1 strategy is present) which we can represent by each of the strategies (see legend on top of Fig. 3).

Figure 3 shows that the 5 behavioral strategies can be clustered into 3 groups: **0**, **4** and the group **R-2-C** that combines all “fair” strategies, i.e. always give 2, reciprocal and compensating strategies. In fact, when error probability $\epsilon =0$ and a risk probability $p=0.9$ (see Fernández Domingos et al^28^ for a deeper analysis on the CRD), strategies **R**, **2** and **C** each occupy around $33\%$ of the stationary distribution (see Markov chain representation in Fig. S28 in the SI), together fully dominating the population, which justifies the very high values of success rate and public account values obtained for error$=0$ in Fig. 2.

It is interesting to note in Fig. 3a–c that although the random drift triad (see Fig. S28) consisting of strategies 2, *R* and *C* is perturbed when errors are present (i.e. allowing for small variations in the stationary distribution of these strategies), the entanglement of their stationary distributions does not collapse with increasing $\epsilon$, as they follow similar trends within each different error type mode. A closer look, comparing delegation and no-delegation, allows one to extract two main insights: in the no-delegation case, even though strategy **0** never dominates the population, it experiences a sharp initial increase with $\epsilon >0$ (Fig. 3a); for the delegation cases, we find that strategy **0** eventually dominates the population by crossing the stationary distributions of the triad **R-2-C** at $\epsilon \sim 0.18$ for program errors (Fig. 3b) and at $\epsilon \sim 0.3$ for delegate errors (Fig. 3c) respectively. The initial sharp increase of the stationary distribution of strategy **0** in Fig. 3a explains the initial faster drop in success rate and public account contributions with $\epsilon$ experienced by the execution errors population when compared with the delegation populations. Since as Fig.3d–i show, success probability and public account values for strategy **0** are equal or close to 0 for low error probabilities, so that a sharp increase in the distribution of strategy **0** greatly harms the overall success rate and average public account values within a population.

Focusing on the success probability as illustrated by Fig. 3d–f, one can see that even though the steepness of the curves of strategies **0** and **4** might change between error types, their plateaus remain at the same values of 0 and 1 respectively. On the contrary, the **R-2-C** triad appears to reach different plateaus for execution and program errors than it does for delegate errors, where the plateau of the latter is higher. Coupling these insights to the already observed trends when analyzing the stationary distributions in Fig. 3a–c, we realize that the intersection point of the program with the execution errors population at $\epsilon \sim 0.16$ in Fig. 2a is mainly dependent on the rise of strategy **0** as the dominate strategy at $\epsilon \sim 0.18$ for the program errors population since there is little difference in success probability of program vs execution errors when comparing Fig. 3a and b. Contrarily, even though strategy **0** becomes dominant in the delegate errors population at $\epsilon \sim 0.3$, overall the success rate of execution errors only surpasses the success rate of delegate errors at $\epsilon \sim 0.45$, because even though strategies **R**, **2** and **C** are not dominant anymore, their success probability is higher in the delegate population than in the execution population as we can observe when comparing Fig. 3a and c.

A similar analysis can be conducted on the average public account values, where one can find that the main driver behind the population averaged values observed in Fig. 2b is the fact that the public account values expected for mono-strategic groups made of strategies **R**, **2** and **C** with execution errors plateau at $C\sim 120$ (Fig. 3g), whereas the same groups with delegation errors (both of the program in Fig. 3h and the delegate type Fig. 3i) reach lower values with increasing error probability. Therefore, even though a sharp initial increase in the distribution of strategy **0** as observed in Fig. 3a might lead to the no-delegation population starting with the lowest group contributions to the public account in Fig. 2b, the increase in error probability and the slower increase of strategy **0** in the other populations will eventually reflect at the population level what is found when comparing the triad **R-2-C** across different error types: no-delegation error types make higher contributions for the majority of the error probability range.

In summary, we find that the high success rates and pro-sociality when there are no mistakes are driven by the stability of a random drift triad formed between strategies **R**, **2** and **C**. With the appearance of errors, independently of their type, this stability is broken, allowing for the rise of the selfish strategy **0** to increase within stationary distribution. A sharp increase for small $\epsilon >0$ in the case of *execution errors* is sufficient to generate the observed qualitative trends described in Fig. 2, where delegation results in better collective outcomes than no-delegation strategies for small error probabilities. But even in the delegation populations represented by *program errors* and *delegate errors*, it is the interplay between the rise of the stationary distribution of **0** in detriment of the stationary distribution of **R**, **2** and **C** that will eventually lead to the decrease of success rate and average public account values to the point that they cross the curves of the *execution errors* population leading to the intersection points marked by dotted-dashed lines in Fig. 2. In this sense, one can conclude that it is the occurrence of errors with a probability $\epsilon >0$ that gives rise to selfish strategies that eventually undermine the collective success and pro-sociality within an evolving population.

### When delegation is optional, adoption rates are higher with pre-set rather than programmable agents

What if the choice to delegate is optional? In this section, the choice to delegate or not is part of the strategy space and we ask in which conditions does delegation dominate the evolving population. As before, an EGT approach is used, wherein delegation and no-delegation strategies compete to determine who will dominate the population in the long-term. In other words, whether the delegation rate, i.e. the sum of the stationary distribution of the delegation strategies as given by Eq. (4) in Methods, is higher than $50\%$, meaning that the population spends more than half of its time in a state associated with a delegation strategy. Moreover, as two delegation approaches have been studied,we will examine here which delegation method will result in higher adoption (delegation) rates in a population in the long-term? Concretely, two hybrid populations will thus be studied, i.e. one where the delegation is implemented through *program errors* and the other where *delegate errors* represent delegation instead. Again, within each population 5 different individual preferences in terms of how individuals would like to react to the behavior of others when playing the CRD (strategies **R**, **C**, **0**, **2** and **4**) are considered, although in this case wanting to delegate (or not), also constitutes part of the player’s strategy when playing the game, resulting in a total of 10 competing strategies within each hybrid population.

**Figure 4:**
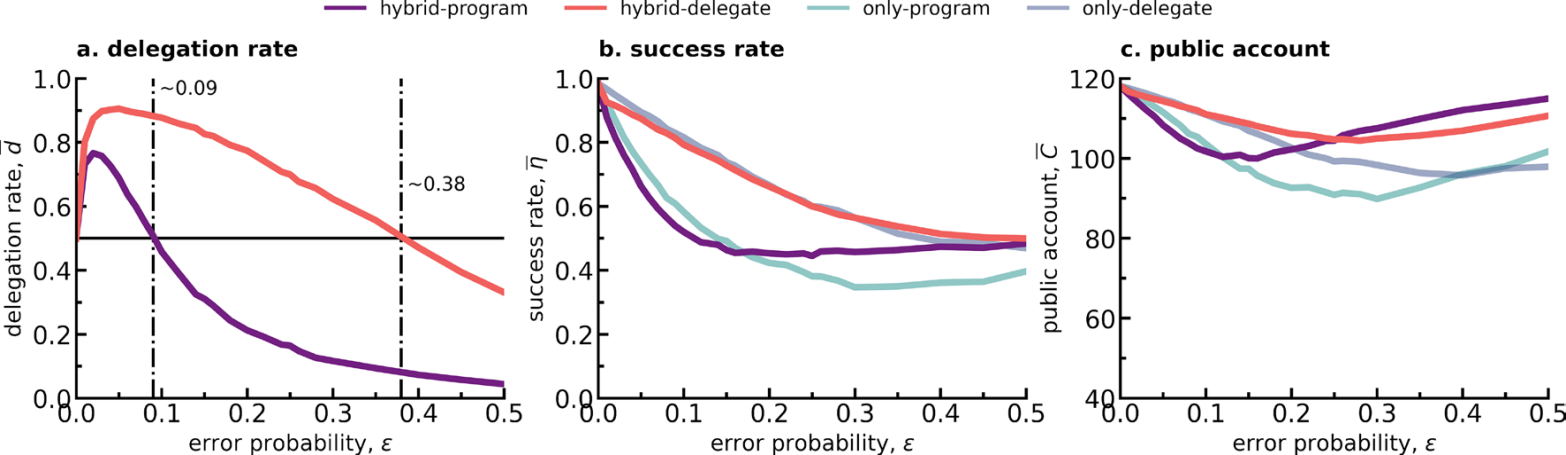
Delegation rate, success rate and average public account in terms of error probability $\epsilon$ for two different hybrid populations where individuals can either adopt a delegation or a no-delegation strategy. In one population the delegation strategy is represented by *program* errors, in the other by *delegate* errors, which are distinguished by color following the legend on the top right corner of the figure, labelled “hybrid-program” and “hybrid-delegate” respectively. The no-delegation strategies are always represented by *execution* errors in this work. Within each population there are therefore 10 competing strategies: *R*, *C*, 0, 2 and 4 for both delegation and the no-delegation case. In (**a**), with a dotted-dashed line we mark the error probability at which the delegation rate drops below 0.5 for each of the shown hybrid populations: at $\epsilon \simeq 0.09$ for *program errors* and at $\epsilon \simeq 0.38$ for *delegate errors*. In (**b**) and (**c**) we reproduce again the results already shown in Fig. 2a and b for the non-hybrid *program errors* and *delegate errors* population, but now in more transparent colors, as indicated in the color legend on top with the labels “only-program” and “only-delegate”. The parameters used to reproduce this figure are: $\beta =0.05$, $Z=100$, $p=0.9$, $r=10$, $E=40$, $A={0, 2, 4}$, $N=6$, $Z=100$, $\#sim=1000$.

Figure 4a shows how delegation rates vary with regards to error probability $\epsilon$ for both an hybrid population where delegation is made through *program errors* and an hybrid population where delegation is represented by *delegate errors* (see Fig. S6 in the SI to consult the same results within the full range $0\le \epsilon \le 1$). With a dashed line we denote when the delegation rate, or in other words, the summed stationary distributions of all delegation strategies within a population as per Eq. (4) given in Methods, drops below 0.5. These are points of interest since they show the boundary condition in terms of error probability at which delegation stops being the most adopted strategic tool within the evolving hybrid population. In summary, the EGT model predicts that delegation rates will be higher when delegation is made through a pre-set agent (*delegate errors* may occur) than by letting individuals program their agents (*program errors* population) for a wide range of error probabilities. This is of special interest since it contradicts the post-experiment survey results that accompanied the experimental study conducted in Fernández Domingos et al.^19^ (with similar game parameters), where participants have answered that they would rather delegate again if they were given the opportunity to program their agents, rather than choose a delegate from a group of pre-set ones. As shown in the SI, the trends observed in the main manuscript remain valid for different values of selection strength $\beta$ (see Figs. S29 and S30, where an increase in selection strength only results in a greater approximation to a step-like function).

In Fig. 4b and c the success rate and average public account values in hybrid populations are plotted in function of the error probability together with the data from Fig. 2a and c, where there were only delegation strategies in the population. While the success rate and average public account values in hybrid populations are a combination of both the delegation and no-delegation strategies, it remains interesting to see how having the choice may improve (or not) the collective decision-making in the CRD. Indeed, even though for small values of $\epsilon$ the difference between success rate and average public account values of the hybrid populations and their non-hybrid counterparts is almost non-existent (with non-hybrid populations fairing slightly better), at a certain value of $\epsilon$, the hybrid population returns higher success rates and contributions to the public account. This effect is especially exacerbated for the case of hybrid populations where delegation is made through *program errors* in both Fig. 4b and c, leading to the conclusion that optional delegation is essential when individuals can program their own agents. When delegation is made through choosing a pre-set delegate (*delegate errors* can be committed), there is little difference between mandatory and optional delegation if one is merely interested in whether the group will achieve success or not.

**Figure 5:**
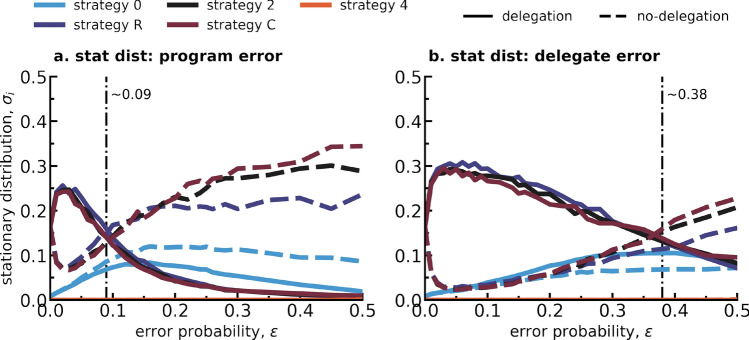
Stationary distribution in terms of error probability $\epsilon$ of the 10 competing strategies present in the hybrid population with *program errors* (**a**) for delegation and in the hybrid population with *delegate errors* (**b**) for delegation. Following the legend on top, each strategy is represented by a color scheme to distinguish between the 5 different conditional behavior preferences, which can then be full lines if belonging to a delegation strategy or dashed lines if to a no-delegation strategy (always referring to individuals who commit *execution errors* in the context of hybrid populations). With a dotted-dashed line at $\epsilon \simeq 0.09$ indicates where delegation rate decreases to $\le 0.5$ in the *program errors* population in panel (**a**); similarly, at $\epsilon \simeq 0.38$ we indicate the error probability at which the *delegate errors* population delegation rate drops below 0.5 - in both cases following what was observed in Fig. 4. The parameters used to reproduce this figure are: $\beta =0.05$, $Z=100$, $p=0.9$, $r=10$, $E=40$, $A={0, 2, 4}$, $N=6$, $Z=100$, $\#sim=1000$.

Figure 5a shows the stationary distribution of the strategies present in the hybrid *program errors* population and Fig. 5b in the hybrid *delegate errors* population (see Fig. S7 in the SI to consult the same results within the full range $0\le \epsilon \le 1$). Similarly to what was previously observed in Fig. 3, the triad of strategies **R**, **C** and **2** appear to dominate the evolution. At error probability $\epsilon =0$ they form a 6-strategies random drift bubble, together making up $99.9\%$ of the total stationary distribution at this point. The double triad then collapses with increasing error probability, firstly leading to an increase in the delegation **R-2-C** triad at the expense of the no-delegation **R-2-C** triad, an interesting phenomenon since here the no-delegation triad appears to inversely follow the delegation one. Eventually the dotted-dashed lines shown in Fig. 4 to mark the moment where the delegation rate drops below 0.5 are again represented, and almost coincident with the inversion of trends between delegation and no-delegation triads **R-2-C**. This switch around $\epsilon \sim 0.09$ for the case of *program errors* and at around $\epsilon \sim 0.38$ for the case of *delegate errors* in Fig. 5 once again reinstates the importance of strategies **R**, **2** and **C** in driving changes in adoption of delegation in hybrid populations: delegation is preferred as long as these three strategies are dominant within the hybrid population when the risk is high. Even though the delegation mechanisms presented in Fig. 5a and b are of different nature, in one case the errors occurs in every parameter while in the other there is a randomization of which wrong agent to pick, it is also relevant to note how the stationary distribution functions of the different strategies appear to follow similar trends, perhaps exhibiting a deeper pattern in how delegation shapes the evolutionary process when in comparison to no-delegation strategies.

## Conclusion

The results discussed above allow us to draw a number of interesting conclusions with regards to the success and delegation rates obtained for different delegation methods. We find that for small error probabilities, our results corroborate previous experimental work^19^ in finding that groups made of individuals that delegate achieve higher success rates in avoiding the collective disaster. Moreover, we find that delegation is not only more successful in solving the dilemma, in the long term it is also the most adopted strategy within a hybrid population where delegation and no-delegation strategies compete with each other. This last insight is also supported by recent work^34^ that links higher delegation rates with decisions that entail a higher risk of loss.

Relating specifically to the problem of solving a CRD with high risk, with this work we find that introducing errors perturbs the random drift triad composed by the three strategies responsible for holding high success rates - reciprocal, compensatory and always-2. All the interesting conclusions, such as the identification of the boundaries when delegation is more successful in solving the dilemma, or when delegation is more adopted within the population, relate to the changes observed in the average stationary distribution of these three strategies. Previous work^19,27,28^ had already identified the importance of these three strategies to reach high levels of success, and this work adds to this literature by showing how the presence of errors perturbs this triad and immediately lowers the expected success rates even for low error probabilities. However, in real-world scenarios mistakes happen, and our work shows that it is better to commit them through an artificial delegate than when implementing the strategy ourselves.

It is relevant to note that in all cases the same error probability $\epsilon$ is being used, both in the delegation and no-delegation scenarios. While each error type may entail different error probabilities or even consider different error probability distributions, there is for now no experimental evidence to support a specific assumption. It is for that reason that the simplest approach of assigning the same probabilities to each of them was chosen in this manuscript, providing thus a proof of concept of our game theoretical delegation model.

Additionally, in the current population model, the principal cannot correct the artificial delegate before the end of the game (the human is thus “outside” and not “on” the loop) nor do the strategies have self-correction mechanisms to identify violations against its encoded behavior. This work is limited to the strategies observed at sufficient frequencies in experimental data^19^. Of course, a wider panoply of strategies can be imagined, for example, but not limited to: memory-n strategies, strategies that self-correct one’s mistakes in the following round, strategies that forgive others mistakes in the previous round, strategies that include a timely revision at a certain point. However interesting, such extensions are left for future work.

In conclusion, to our knowledge, it is the first time that a delegation mechanism is formalized without having to rely on explicit differences in the behavioral strategies as, for instance, associated delegation costs^25^ or self-interested agents that specifically play a delegation game^35^. This contribution is very important, as often, in both the experimental settings or real-world applications, the main difference between delegated and non-delegated action lies in the moment when the commitment is made, which in this modeling work we connect with the moment when errors might be committed.

In order to align human expectations with artificial delegates behavior, it is of utmost importance to first understand how wide the alignment gap actually is. This work analyzes how committing mistakes in the programming of an agent (or the mistakes in selecting an agent) can influence the macro-behavior of groups in mixed-motive situations, when these interact in society on behalf of their users. In this sense, it provides a stepping stone in how to address the AI alignment problem^10,11^: by showing how misalignment affects decision-making, providing a framework for thought experiments and mechanism testing that aim to resolve the issue.

## Methods

### Collective-risk dilemma game

The collective-risk dilemma (CRD) game^19,26,27^ is used to frame the interactions in this work. The game is played in groups of *N* players, each starting with an endowment of *E* monetary units. The players have *r* rounds to contribute to a public account a value between 0 and *E*/*r* (and this action space *A* might be more or less granular^36^). If the group is able to collectively accrue at least $E/2\times N$ within the public account by the end of the last round, everyone is able to keep whatever remains in their personal endowments. Otherwise, there is a risk probability *p* of everyone losing it all. Similarly to^19,26^, if the threshold is met before the end of the *r* rounds, the game stops so that the Public Account values will rarely accrue more than the set threshold $E/2\times N$. The payoff $\Pi$ collected by a player *i* after playing the CRD is given by Eq. (1), where $c_{ik}$ represents the contribution given by a player *i* on round *k* of the game and $\theta (x)$ is the Heaviside function ($\theta (x)=1$ if $x\ge 0$ and $\theta (x)=0$ otherwise).

1 $$\begin{aligned} \Pi _i = \left( E - \sum _{k=1}^r c_{ik}\right) \left[ p\theta \left( \sum _{j=1}^N\sum _{k=1}^r c_{jk} - E/2\times N\right) + (1-p)\right] \end{aligned}$$

### Strategy and errors definition

In this modelling work, the whole spectrum of player’s true preferences are assumed to correspond to the same behavioral diversity as the one previously experimentally explored in Fernández Domingos et al.^19^, where in the delegation treatment, participants were given a pool of 5 pre-set agents to choose from to play the CRD: always-0, always-2, always-4, reciprocal and compensatory. The first 3 behavioral profiles correspond to fixed strategies where the agents contribute in every round 0, 2 or 4, respectively. The last two behavioral profiles correspond to conditional behaviors, where the reciprocal strategy corresponds to contributing 0, 2 and 4 given that the rest of the group members have contributed on average in the previous round 0, 2 or 4, hence reciprocating their behavior in the following round; while the compensatory strategy on the contrary contributes 4, 2 and 0 if the others have contributed 0, 2 or 4 on average in the previous round, therefore compensating their contributions. Both conditional behavioral profiles are assumed to contribute 2 in the first round when there is no previous round to condition its behavior and all players are assumed to stop contributing once the collective target is reached.

The individuals within the evolving population are assumed to make mistakes with a certain probability $\epsilon$ while executing their strategies. The way we distinguish between delegated and non-delegated action lies precisely in the moment on which the individuals make those mistakes. Here, we consider that an individual playing on their own might deviate from their intended strategy in each round of play, committing what we may call *execution errors*. If on the contrary, the individual delegated the strategy to an autonomous agent, the latter is assumed to not commit any execution errors during the game-play. However, the principal is considered to be human, and is therefore bounded by the same probability $\epsilon$ of committing mistakes. So in the case of the delegated action, we assume the mistakes are committed in the choice or the programming of the autonomous agent. For example, if the protocol is for the human to choose from a set of 5 agents and they wrongly choose an agent that actually does not correspond to their behavior profile (they are reciprocal but choose always-2 by mistake), we call this a *delegate error*. Following^19^, another possibility is for the human principal to program their own agents, in which case a human principal who is reciprocal might commit a *program error* and code their agent to contribute 4 (instead of 0) if the others in the group have contributed 0 in the previous round. Note that, when an individual delegates and therefore commits an error with probability $\epsilon$, this might not correspond directly to the probability of deviating from the course of action that would instead be implemented by the same strategy when $\epsilon =0$ (in other words, the actual error rate). However, as shown by Fig. S31 in the SI where we compute actual error rate vs. error probability $\epsilon$, the strategies reciprocal, compensatory and always-2 in the case of delegate errors constitute the only cases where actual error rate greatly deviates from the error probability. This reiterates our design choice for the different error modes, as program errors behave very similarly to execution errors when we assess their actual error rate, but follow instead the curves of delegate errors when we look at the main Figs. 2 and 3 of the Results section.

In essence, our model considers 5 different behavioral profiles and 3 different error modes. The 5 different behavioral profiles follow a program with 4 different settings: one setting to define the action at the first round, and 3 settings to define how much to contribute if others have contributed 0, 2 or 4 in the previous round (we assume at each round, participants can either contribute 0, 2 or 4). Each agent therefore always has access to the total contributions made in the previous round and to their own contribution (which together allows the agent to compute the average of others in the previous round), as well as whether the group has already reached the threshold or not (which indicates to the agent when to stop playing the game/contributing to the public account). The 3 different error modes represent non-delegation action (through *execution errors*) and two different modes of delegated action (*delegate error* and *program error*). Even though we consider a universe of 15 different strategies, depending on the specific research question, the evolving population might not contain all the 15 competing against each other. The number of strategies present within a given evolving population is given by *S*, for example Figs. 2 and 3 compare results between populations where the $S=5$ behavioral profiles compete but only one error mode is present; whilst Figs. 4 and 5 consider hybrid populations where 5 behavioral profiles and 2 error modes compete to study the evolution of delegation rate, hence $S=10$ strategies are present.

### Evolutionary game theory and the small mutation limit

As mentioned in the Introduction, an EGT framework^20–22,32,33,37–39^ is used to understand how delegation to autonomous agents affects (human) behavior in collective social dilemmas in the long term. In summary, such an approach consists of analytically solving or numerically simulating the evolution of a population of *Z* individuals that play a game between themselves according to their assigned strategies. In order for an individual’s strategy to propagate within the population, it must harness more fitness than the others. A strategy’s fitness corresponds to the expected payoff a certain strategy gets if it plays the CRD within all possible different group configurations (consisting, or not, all the other strategies available) within that population. Once a round of play is completed, individuals are randomly chosen to update their strategy by either imitating a better co-player or by randomly changing their strategy to one of those possible. In the case of imitation, another individual is randomly selected from the population, and its strategy is more likely to be imitated if its payoff is higher than the current strategy of the focal agent chosen to update. This likelihood of imitation is further tuned by a selection strength parameter $\beta$ which is part of the Fermi-function used in the stochastic evolutionary model^20,21,32,33,38,39^. Our model specifically considers stochastic strategies (see subsection Strategy definition above), in which case, the strategy actually played by each individual might not correspond to the originally intended strategy that is part of the set of competing strategies within the population. We want to highlight that at the moment of imitation learning , an individual will only imitate the originally intended strategy of another - apparently more successful - individual, similarly to what is done in^7,30,31^. Similarly, if an individual suffers mutation, the new strategy should come from within the set of co-evolving strategies considered without the effects of its possible stochasticity.

The evolutionary process described allows us to calculate the stationary distribution, $\sigma$, of each competing strategy within an evolving population playing the CRD. The stationary distribution will mirror the long term success of the strategies relative to each other within this CRD’s context, informing us about which ones will prevail if enough time is provided for evolution to reach a stable solution. Given that we will always have at least 5 competing strategies within a population we take the small mutation limit^22,32,33^ approach to facilitate our analysis. In this case, we assume that the probability of an individual to adopt another strategy through mutation (rather than imitation) is so small that the population spends most of its time in a monomorphic state. When a new strategy appears through mutation, the new strategy either takes over the population or disappears long before another mutation appears. This way, the evolutionary dynamics can be approximated through a Markov chain with the number of states equal to the number of strategies.

Our implementation^40^ uses version v0.1.12 of the recently published hybrid C++/Python library EGTtools^22,41^, whose methods allows us to easily retrieve the stationary distribution provided the game payoffs associated with each strategy and group composition and the relevant population parameters such as the selection strength $\beta$ and the number of individuals *Z*. Given the noisy nature of the model in analysis, the game payoffs have to be previously estimated through a series of simulated plays within each group composition before being used as input to the small mutation limit methods described in Fernández Domingos et al.^22^, so that the number of simulations $\#sim$ used to make this estimation is also a relevant parameter to reproduce the results exhibited in the Sect. "Results and discussion".

### Relevant group metrics in the CRD

With the stationary distribution, we are then able to calculate game-related metrics that are relevant for our analysis in a time-independent way. We calculate the probability of a group made of *N* players of each strategy achieving success in the CRD, which will be denoted by $\eta _i$ (for each strategy *i*). A weighted average of that quantity with each strategy’s stationary distribution will then return the average success rate^27,42^, $\overline{\eta }$, associated with a population where those strategies are present, as shown in Eq. (2).

2 $$\begin{aligned} \overline{\eta } = \sum _{i}^{S} \sigma _i \eta _i \end{aligned}$$

Another metric of interest that informs us on the overall pro-sociality observed within a given population is its average public account $\overline{C}$. In order to calculate that, we consider the stationary distribution of the different competing strategies and the amount that a monomorphic group made up of *N* individuals of a given strategy contributes in total (of all rounds *r*) to the public account, $C_i$ for each strategy *i*. Analogously to the calculation used to determine the success rate of a population, a weighed average given by Eq. (3) determines $\overline{C}$.

3 $$\begin{aligned} \overline{C} = \sum _{i}^{S} \sigma _i C_i \end{aligned}$$

By calculating these quantities for both a population where only no-delegation is allowed and a population where only delegation is allowed, we can then infer which would be the best long-term solution for the CRD for any combination of other parameters, for example our parameter of interest, the probability of error (which is precisely what we demonstrate in the Results section). Note that given the stochastic nature of quantities aforementioned, $\eta$ and *C* are themselves the result of an average of values calculated over a given number of computer simulations $\#sim$ that consider the probabilistic nature of the error.

If instead of populations where only delegation or no-delegation strategies are considered, we can instead explore a hybrid population where both delegation and no-delegation strategies are possible. In this case, we are also able to define a delegation rate $\overline{d}$, by summing over the stationary distributions associated with delegation strategies (subset $S_{del}$) only to examine the prevalence of delegation within such a hybrid population, as shown in Eq. (4). Such a method allows us to understand when does delegation become dominant in a population where it is optional, for example, when $\overline{d}>0.5$, as shown in the section Results.

4 $$\begin{aligned} \overline{d} = \sum _{s \in S_{del}} \sigma _s \end{aligned}$$

### Supplementary Information

Supplementary Figures.

## Data Availability

All data generated or analysed during this study are included in this published article and its supplementary information files. All code to produce said data is available at: https://doi.org/10.5281/zenodo.10392017^40^. No experiments with human participants were performed in this work.
