# Supplementary material for: Committing to the wrong artificial delegate in a collective-risk dilemma is better than directly committing mistakes
Source: Sci Rep. 2024 May 7;14:10460. doi: 10.1038/s41598-024-61153-9 (PMC11076577; doi:10.1038/s41598-024-61153-9)
Supplement: Supplementary file 1 — Supplementary Figures. [file 41598_2024_61153_MOESM1_ESM.pdf]

Supplementary Information - Committing to the wrong artificial delegate in a collective-risk dilemma is better than directly committing mistakes

Inês Terrucha, Elias Fernández Domingos, Pieter Simoens, Tom Lenaerts

Supplementary Figures

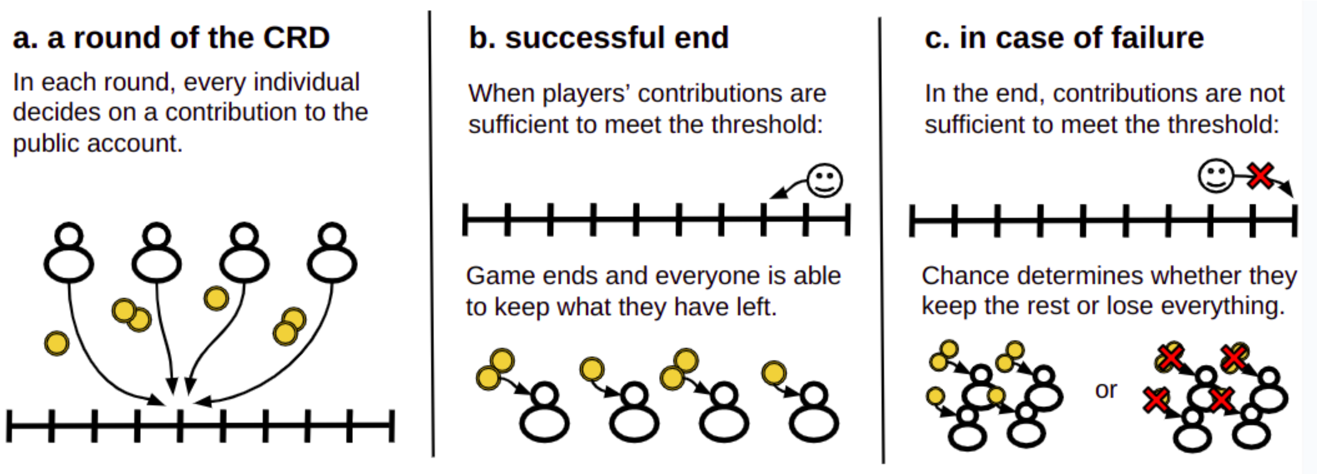

Supplementary Figure S1. Illustration of the timeline of a CRD game. (a.) shows that in every round, all group members decided how much to contribute from their private endowments to the public account. The game may end in two scenarios: (b.) the group is successful in meeting the collective threshold and everyone may keep the remainder of their endowments (this may occur before the last round is played) or (c.) after the last round is played the group still has not been able to meet the collective threshold, so that, with a certain risk probability, the group faces collective total loss of the remainder of their endowments.

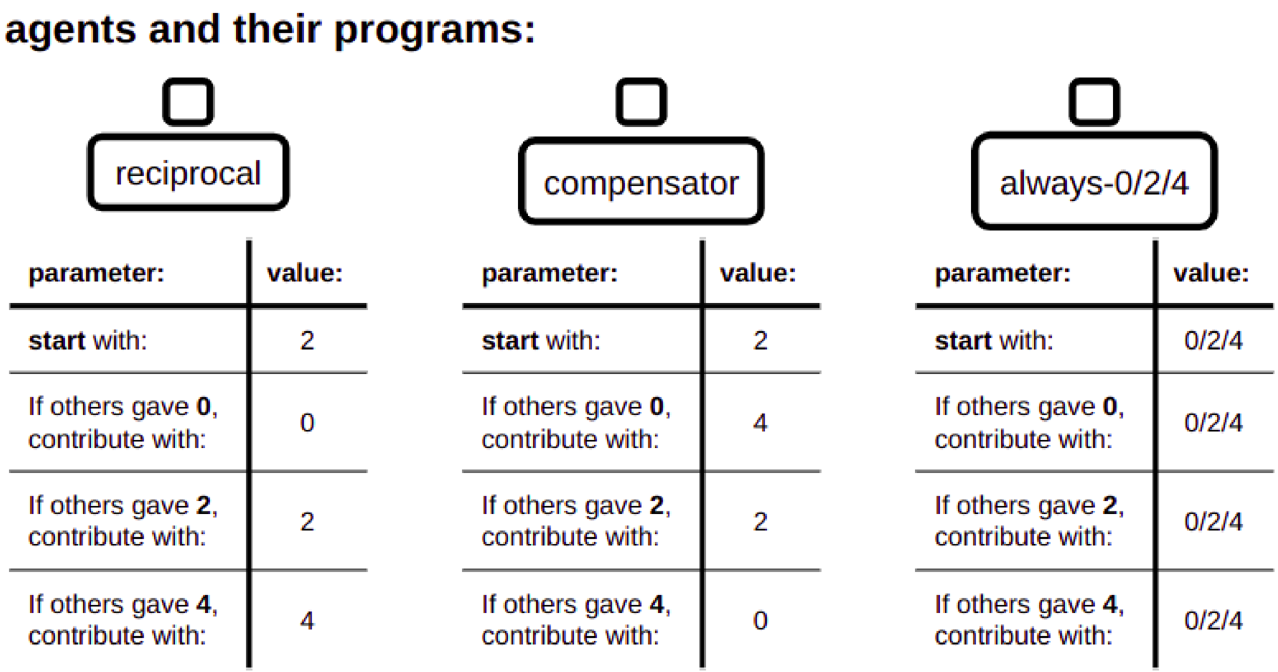

Supplementary Figure S2. Simple programs that represent the set of 5 behavioral profiles considered in this work. On the left, the program corresponding to the "reciprocal" profile, where the agent/individual will start the game by contributing with 2 and then will reciprocate whatever the others have contributed in the previous round. In the middle, the program corresponding to the "compensator" profile, where the agent/individual will start the game by contributing 2 and then will compensate what the others have contributed in the previous round by choosing the highest action if the others contributed the lowest, the lowest if others contributed the highest and 2 if others also previously contributed 2. Finally, on the right column, a representation of the fixed behavioral profiles "always-0", "always-2" and "always-4", where respectively the agent/individual will contribute 0, 2 and 4 independently of how others behaved in the previous round. This image also illustrates for the reader the relation between how others played in the previous round might influence the next action of the agent. Note that the agent takes into account the actions of the previous round taken by the other group members averaged and rounded to the nearest action within the set of possible actions, in this case the illustration is shown for the 3 action scenario with $S = 0, 2, 4$.

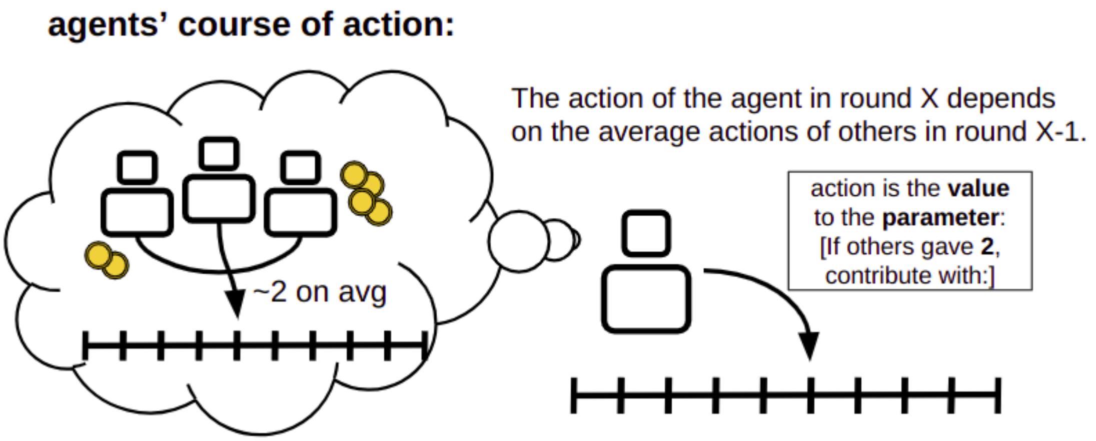

Supplementary Figure S3. Example of the simple program behind every individual's strategy as implemented within this work. An agent (either a delegate or the individual itself), will take an action in round X by considering how much the others have contributed (on average and rounded to the closest possible action considered, in this case belonging to the set $S = 0, 2, 4$) in round $X - 1$. Specifically, if others have contribute on average 2, the agent will take an action corresponding to the value of the parameter "If others gave 2, contribute with:" as configured in the strategy that the agent is playing with (unless it is the case of no-delegation, in which case an error may occur between the selection of the action by the strategy and the implementation of the strategy). Note that in the case of delegation, the strategy that the agent is playing with might not correspond to the action that the original strategy (before an error occurred in delegation) would have implemented.

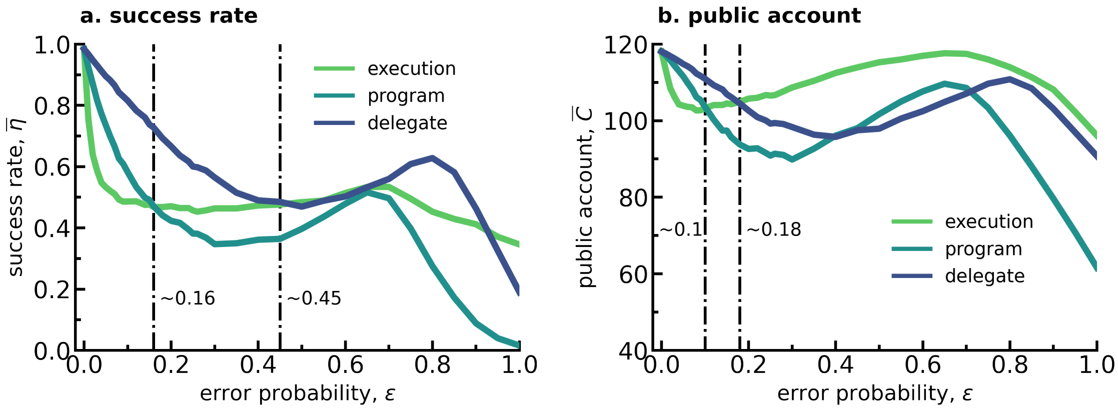

Supplementary Figure S4. Success rate and average public account values observed in 3 populations distinguished by the type of errors their individuals commit. Equivalent to Fig. 1 in the main text, but here for all the range $0 \leq \varepsilon \leq 1$. Following the color legend inside each sub-figure, the errors can be of type *execution*, *program* and *delegate*. Sub-figure **a.** refers to success rate in terms of error probability ε. In **a.**, two dotted-dashed lines at $x \sim 0.16$ and $x \sim 0.45$ indicate the value of ε until which the program and the delegate population (both representing a type of delegation), respectively, achieve higher success rates than the execution (no-delegation) population. Sub-figure **b.** refers to (average) public account in terms of error probability ε. Again in **b.**, with two dotted-dashed lines we indicate the value of ε until which the program ($\varepsilon \sim 0.1$) and the delegate population ($\varepsilon \sim 0.16$) constitute more pro-social groups (that on average contribute more to the public account) than the execution population. To produce this figure, the following parameters were used: $\beta = 0.05$, $Z = 100$, $\#sim = 10000$, $p = 0.9$, $r = 10$, $E = 40$, $A = 0, 2, 4$, $N = 6$.

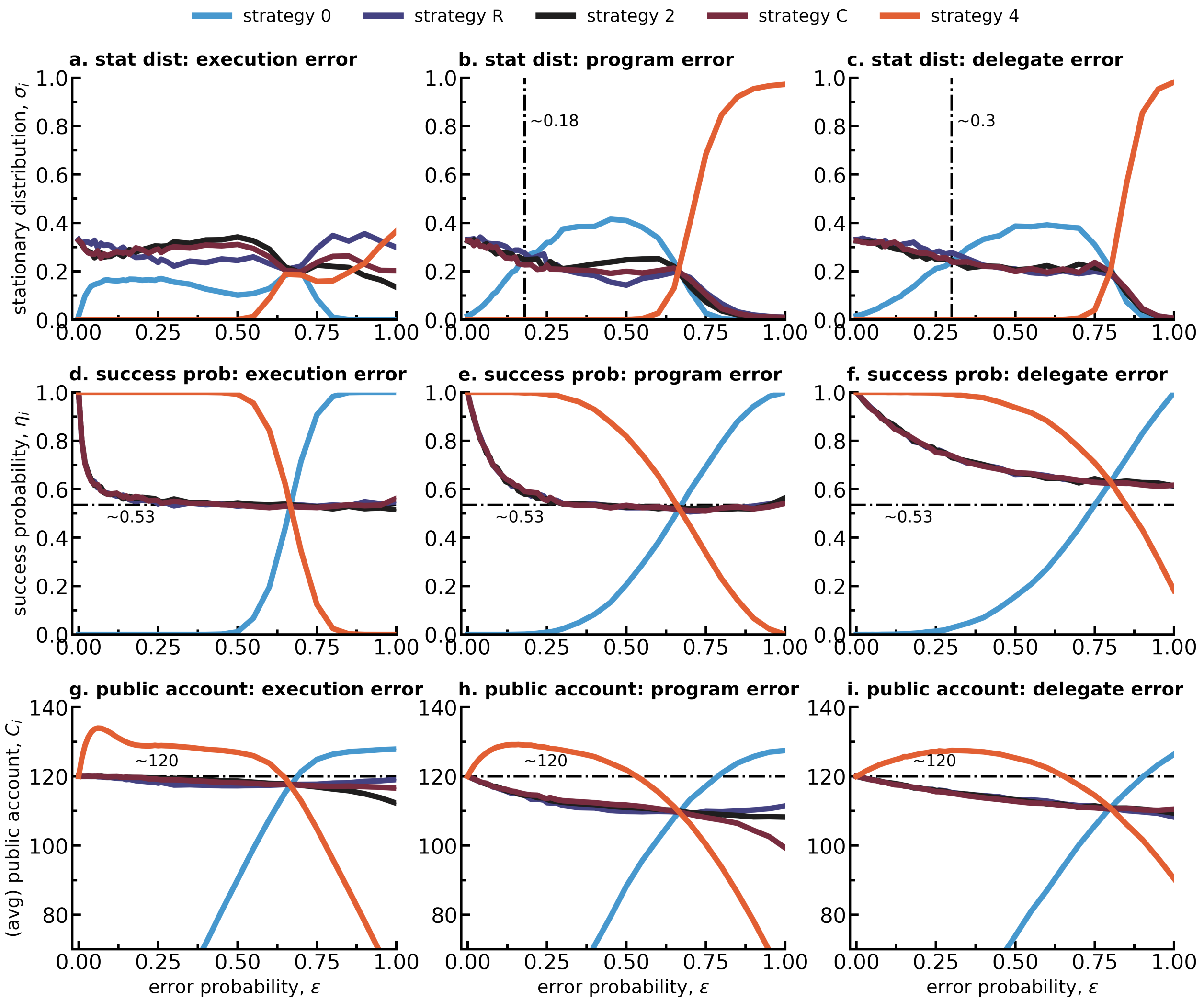

Supplementary Figure S5. Stationary distribution, success probability and average public account per strategy present in a given error type population. Equivalent to Fig. 2 in the main text, but here for all the range $0 \leq \varepsilon \leq 1$. Columns show the different quantities for each error type population: first column execution errors, middle column for program errors and last column for delegate errors. Rows (and y-axis) represent each different quantity of interest: first row shows stationary distribution, second and last show respectively success probability and average public account per each strategy's monomorphic group as a result of $\#sim = 10000$ simulated rounds of play. All quantities are plotted in terms of error probability ε. In **a.** we can observe that strategies *2*, *R* and *C* dominate for every ε even though *0* has a sharp increase immediately when $\varepsilon > 0$. In **b.** and **d.** we denote with a dotted-dashed line at $\varepsilon \sim 0.18$ and $\varepsilon \sim 0.3$ when strategy *0* becomes the most dominant strategy by surpassing *2*, *R* and *C*. In **d.**, **e.** and **f.** with an horizontal dotted-dashed line we mark the success probability value observed in populations with execution error (**d.**) at $\varepsilon = 0.5$ to facilitate comparison between sub-figures. Similarly, in **g.**, **h.** and **i.** we use an horizontal dotted-dashed line to denote the public account threshold (and therefore most efficient group contribution) of $C = 120$. This figure was produced with the same parameters as Fig. 4: $\beta = 0.05$, $Z = 100$, $\#sim = 10000$, $p = 0.9$, $r = 10$, $E = 40$, $A = 0, 2, 4$, $N = 6$.

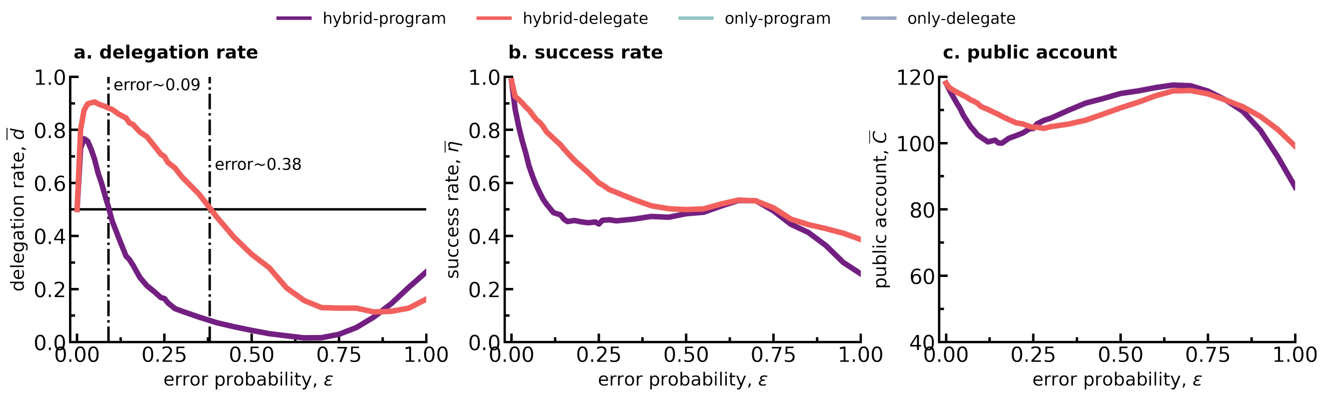

Supplementary Figure S6. Delegation rate, success rate and average public account in terms of error probability ε for two different hybrid populations where individuals can either adopt a delegation or a no-delegation strategy. Equivalent to Fig. 3 in the main text, but here for all the range $0 \leq \varepsilon \leq 1$. In one population the delegation strategy is represented by *program* errors, in the other by *delegate* errors, which are distinguished by color following the legend on the top right corner of the figure, labelled "hybrid-program" and "hybrid-delegate" respectively. The no-delegation strategies are always represented by *execution* errors in this work. Within each population there are therefore 10 competing strategies: R, C, 0, 2 and 4 for both delegation and the no-delegation case. In a., with a dotted-dashed line we mark the error probability at which the delegation rate drops below 0.5 for each of the shown hybrid populations: at $\varepsilon \simeq 0.09$ for *program errors* and at $\varepsilon \simeq 0.38$ for *delegate errors*. In b. and c. we reproduce again the results already shown in Fig. 4a. and b. for the non-hybrid *program errors* and *delegate errors* population, but now in more transparent colors, as indicated in the color legend on top with the labels "only-program" and "only-delegate". The parameters used to reproduce this figure are: $\beta = 0.05$, $Z = 100$, $p = 0.9$, $r = 10$, $E = 40$, $A = 0, 2, 4$, $N = 6$, $Z = 100$, $\#sim = 1000$.

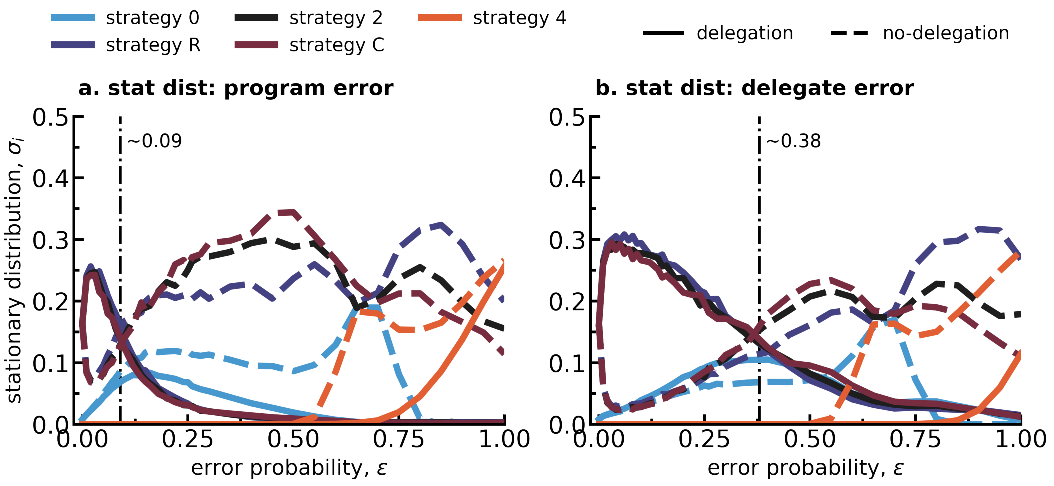

Supplementary Figure S7. Stationary distribution in terms of error probability ε of the 10 competing strategies present in the hybrid population with *program errors* (a.) for delegation and in the hybrid population with *delegate errors* (b.) for delegation. Equivalent to Fig. 4 in the main text, but here for all the range $0 \leq \varepsilon \leq 1$. Following the legend on top, each strategy is represented by a color scheme to distinguish between the 5 different conditional behavior preferences, which can then be full lines if belonging to a delegation strategy or dashed lines if to a no-delegation strategy (always referring to individuals who commit *execution errors* in the context of hybrid populations). With a dotted-dashed line at $\varepsilon \simeq 0.09$ indicates where delegation rate decreases to ≤ 0.5 in the *program errors* population in panel a.; similarly, at $\varepsilon \simeq 0.38$ we indicate the error probability at which the *delegate errors* population delegation rate drops below 0.5 - in both cases following what was observed in Fig. 6. The parameters used to reproduce this figure are: $\beta = 0.05$, $Z = 100$, $p = 0.9$, $r = 10$, $E = 40$, $A = 0, 2, 4$, $N = 6$, $Z = 100$, $\#sim = 1000$.

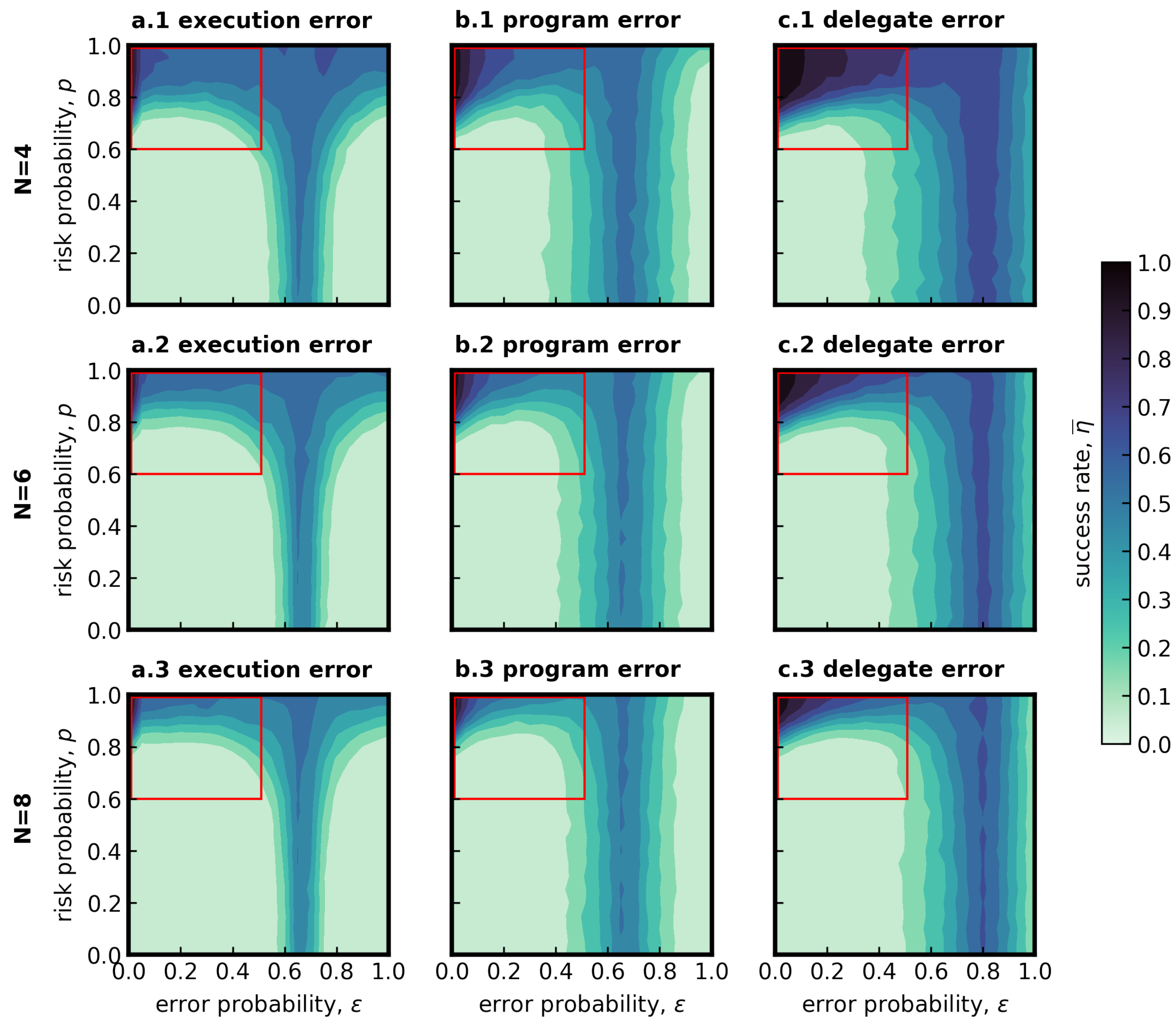

Supplementary Figure S8. Success rate (following the color legend on the right of the figure) for different **group sizes** N in terms of risk (y-axis) and error probability (x-axis). The image was produced for $\beta = 0.05$, $Z = 100$, $r = 10$ rounds and an action space of 3 actions ($A = \{0, 2, 4\}$), following the same variables used in the images featuring the main text. With a red square we denote the region of interest to this study: high risk and low error probability. With this image we conclude that high risk is of interest since it is where the effect of the type of error is most observed. Even though we plot for all error probabilities for analytical analysis, we consider it unlikely that human error would occur with a frequency higher than 0.5 without it being intentional rather than a mistake in the decision-making process.

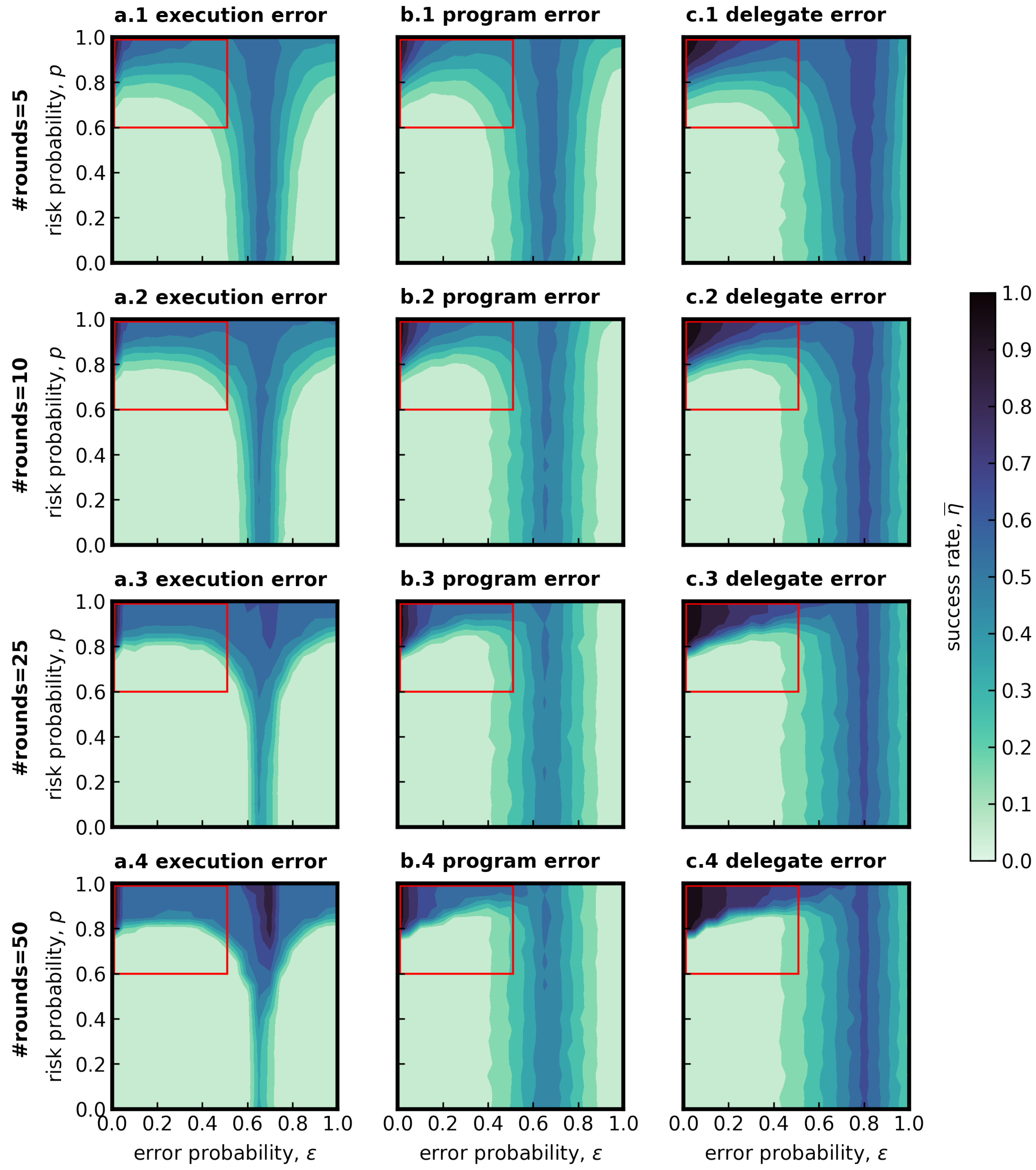

Supplementary Figure S9. Success rate (following the color legend on the right of the figure) for different **number of rounds** r in terms of risk (y-axis) and error probability (x-axis). The image was produced for $\beta = 0.05$, $Z = 100$, group size $N = 6$ and an action space of 3 actions ($A = \{0, 2, 4\}$), following the same variables used in the images featuring the main text. With a red square we denote the region of interest to this study: high risk and low error probability. Again, we see that high risk is of interest since it is where the effect of the type of error is most observed. Even though we plot for all error probabilities for analytical analysis, we consider it unlikely that human error would occur with a frequency higher than 0.5 without it being intentional rather than a mistake in the decision-making process.

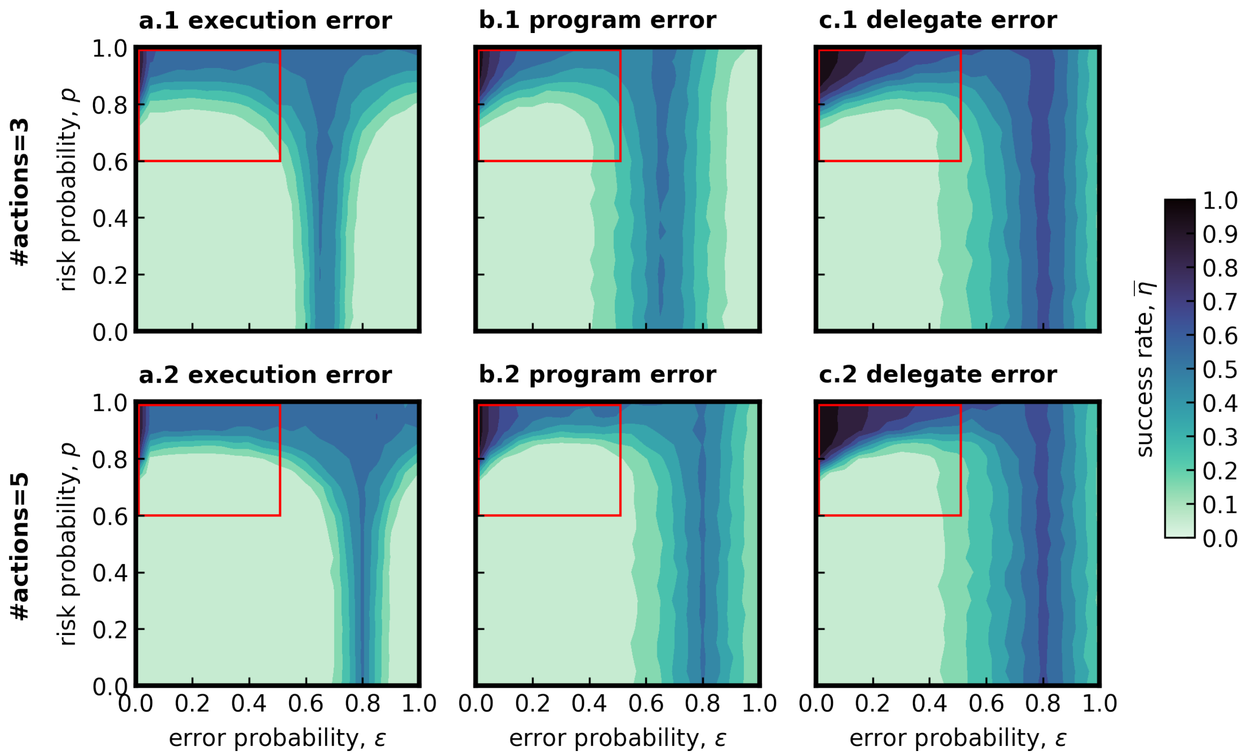

Supplementary Figure S10. Success rate (following the color legend on the right of the figure) for different **action space granularity** ($\#actions = 3$ when $A = \{0,2,4\}$ and $\#actions = 5$ when $A = \{0,1,2,3,4\}$) in terms of risk (y-axis) and error probability (x-axis). The image was produced for $\beta = 0.05$, $Z = 100$, $r = 10$ rounds and group size $N = 6$, following the same variables used in the images featuring the main text. With a red square we denote the region of interest to this study: high risk and low error probability. Again, we see that high risk is of interest since it is where the effect of the type of error is most observed. Even though we plot for all error probabilities for analytical analysis, we consider it unlikely that human error would occur with a frequency higher than 0.5 without it being intentional rather than a mistake in the decision-making process.

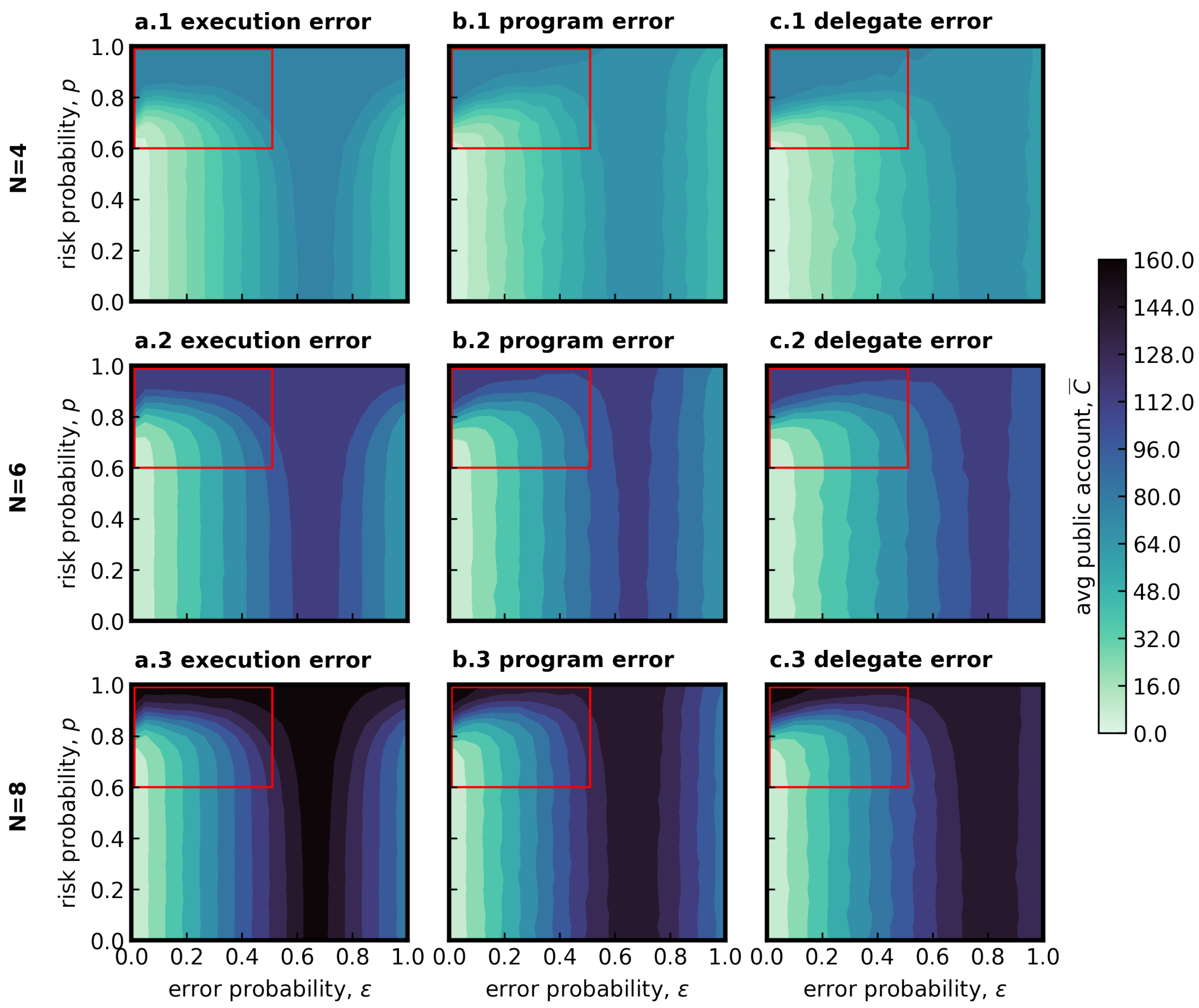

Supplementary Figure S11. Average public account (following the color legend on the right of the figure) for different **group sizes** N in terms of risk (y-axis) and error probability (x-axis). The image was produced for $\beta = 0.05$, $Z = 100$, $r = 10$ rounds and an action space of 3 actions ($A = \{0, 2, 4\}$), following the same variables used in the images featuring the main text. With a red square we denote the region of interest to this study: high risk and low error probability. Again, we see that high risk is of interest since it is where the effect of the type of error is most observed. Even though we plot for all error probabilities for analytical analysis, we consider it unlikely that human error would occur with a frequency higher than 0.5 without it being intentional rather than a mistake in the decision-making process.

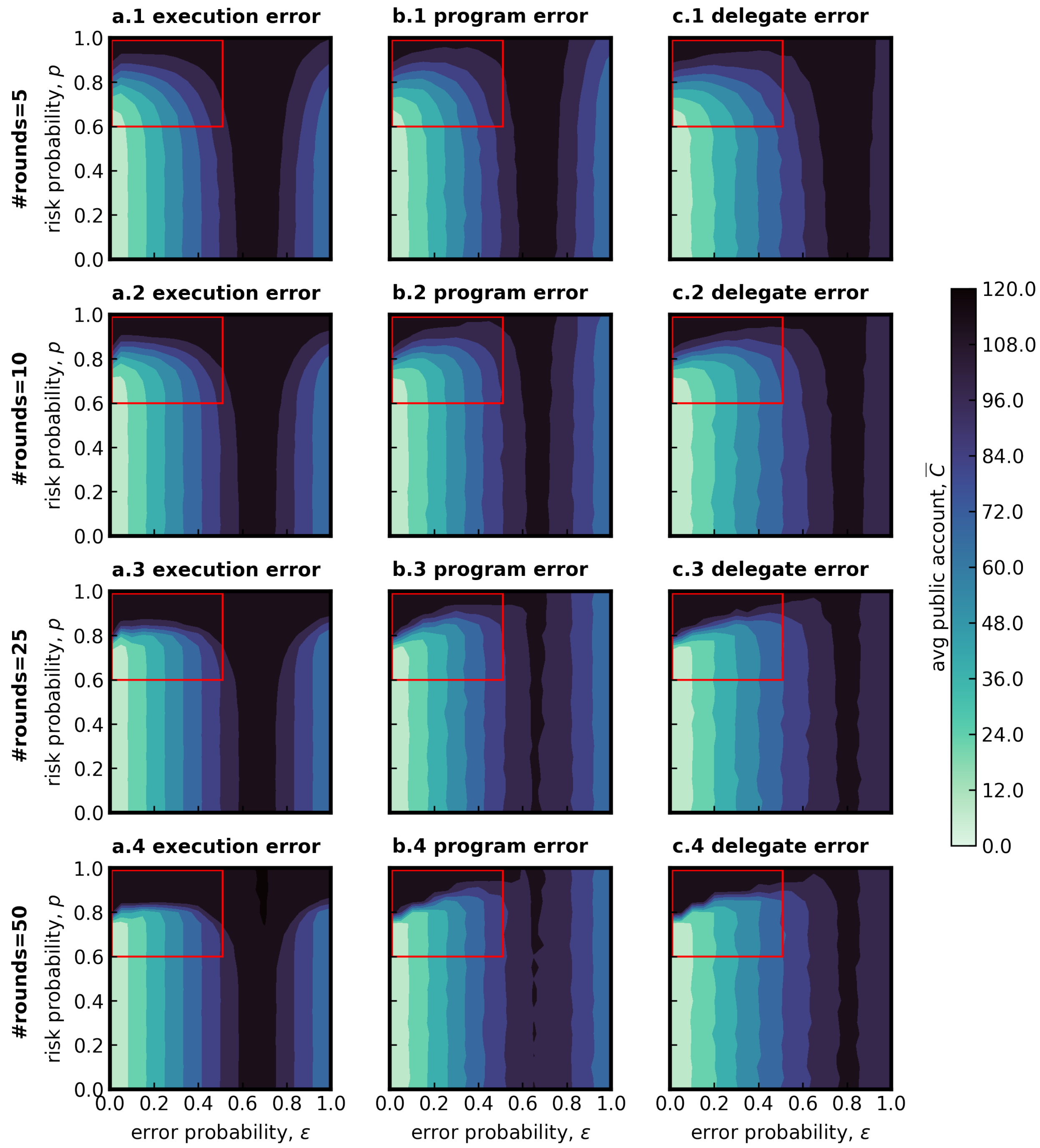

Supplementary Figure S12. Average public account (following the color legend on the right of the figure) for different **number of rounds** r in terms of risk (y-axis) and error probability (x-axis). The image was produced for $\beta = 0.05$, $Z = 100$, group size $N = 6$ and an action space of 3 actions ($A = \{0, 2, 4\}$), following the same variables used in the images featuring the main text. With a red square we denote the region of interest to this study: high risk and low error probability. Again, we see that high risk is of interest since it is where the effect of the type of error is most observed. Even though we plot for all error probabilities for analytical analysis, we consider it unlikely that human error would occur with a frequency higher than 0.5 without it being intentional rather than a mistake in the decision-making process.

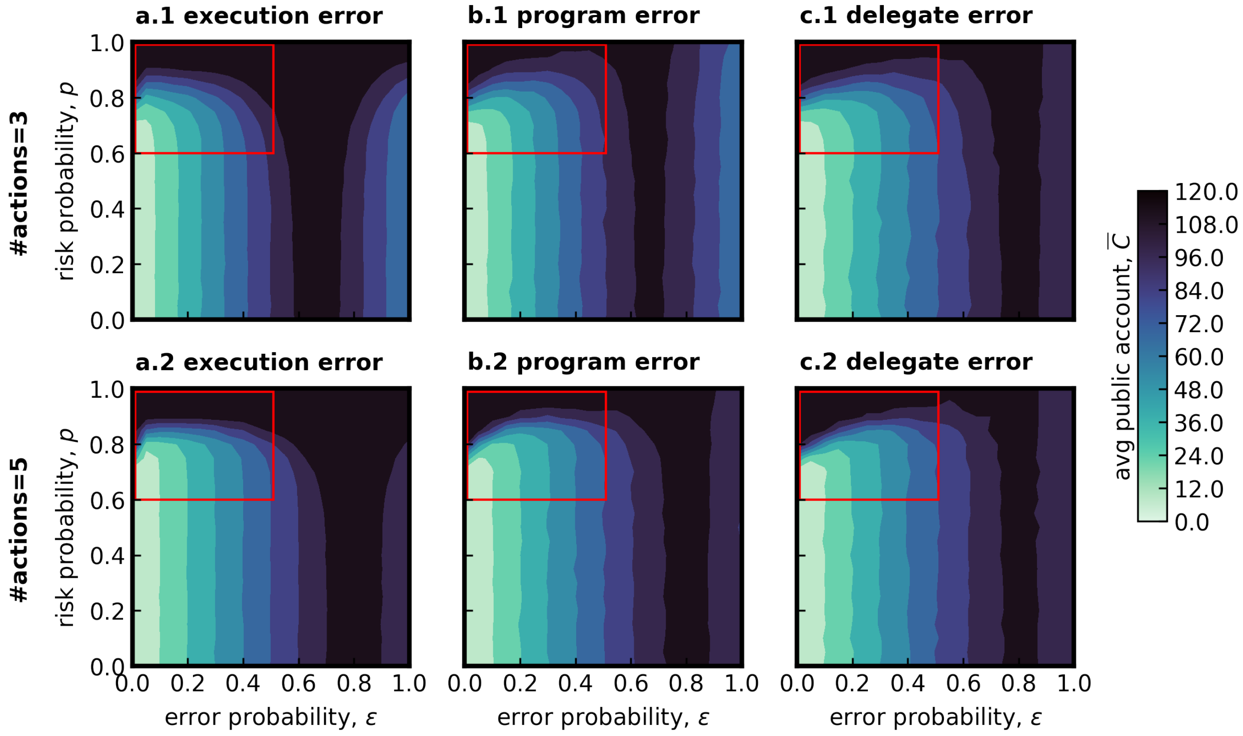

Supplementary Figure S13. Average public account (following the color legend on the right of the figure) for different **action space granularity** ($\#actions = 3$ when $A = \{0,2,4\}$ and $\#actions = 5$ when $A = \{0,1,2,3,4\}$) in terms of risk (y-axis) and error probability (x-axis). The image was produced for $\beta = 0.05$, $Z = 100$, $r = 10$ rounds and group size $N = 6$, following the same variables used in the images featuring the main text. With a red square we denote the region of interest to this study: high risk and low error probability. Again, we see that high risk is of interest since it is where the effect of the type of error is most observed. Even though we plot for all error probabilities for analytical analysis, we consider it unlikely that human error would occur with a frequency higher than 0.5 without it being intentional rather than a mistake in the decision-making process.

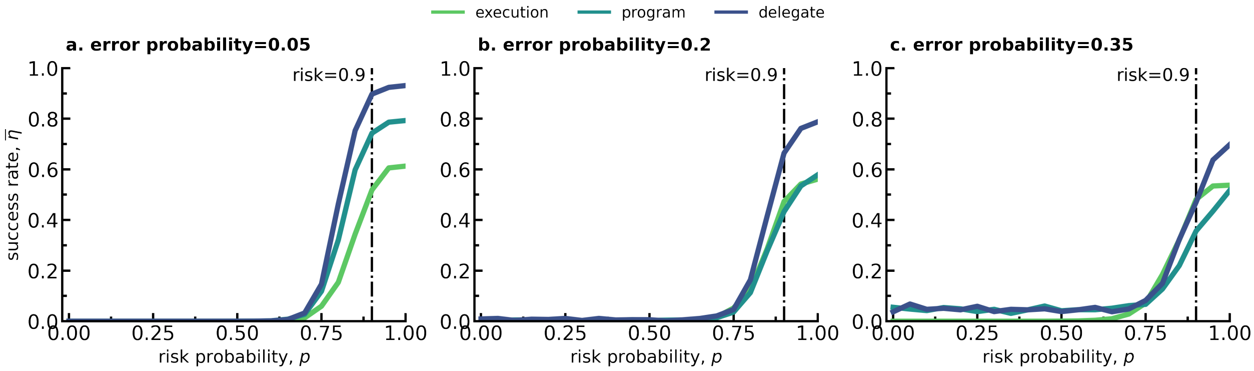

Supplementary Figure S14. Focus on success rate in terms of risk for three different fixed error probabilities ($\varepsilon = 0.05$, $\varepsilon = 0.2$ and $\varepsilon = 0.35$ as expressed on each panel subtitle). Different curves show how the success rate varies for each error type - execution, program or delegate - shown in the figure legend on top. Other parameters used to reproduce the figure were: $\beta = 0.05$, $Z = 100$, group size $N = 6$, action space with $\#actions = 3$ and total number of rounds $r = 10$ following the parameters used in the main text figures. Moreover, we mark with a dashed-dotted line when risk is 0.9, the value used to reproduce the images in the main text. As previous work has already shown, higher risk leads to higher success following a step-like function. Here we show how higher risk is associated with a bigger difference (for lower risk probability there is almost no effect of delegation in success rates) in success rate when we compare between delegation and no-delegation. As also shown in the main text, this difference is also dependent on error probability.

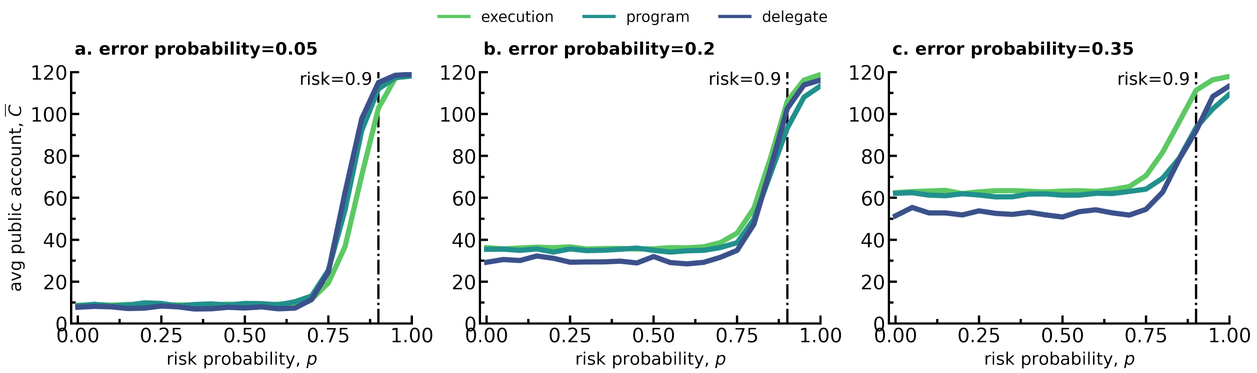

Supplementary Figure S15. Focus on average public account in terms of risk for three different fixed error probabilities ($\varepsilon = 0.05$, $\varepsilon = 0.2$ and $\varepsilon = 0.35$ as expressed on each panel subtitle). Different curves show how the average public account varies for each error type - execution, program or delegate - shown in the figure legend on top. Other parameters used to reproduce the figure were: $\beta = 0.05$, $Z = 100$, group size $N = 6$, action space with $\#actions = 3$ and total number of rounds $r = 10$ following the parameters used in the main text figures. Moreover, we mark with a dashed-dotted line when risk is 0.9, the value used to reproduce the images in the main text. Unlike success rate, lower risk probabilities also result in a difference in average public account values observed between different delegation modes.

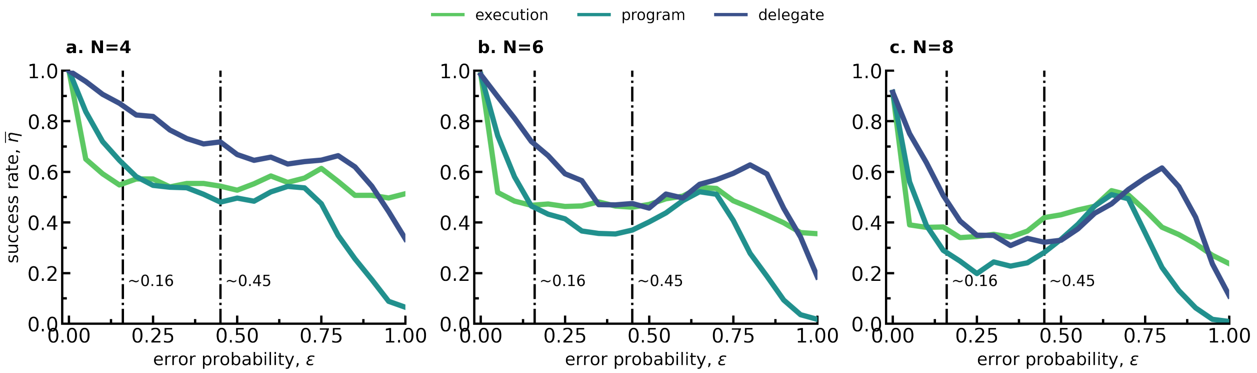

Supplementary Figure S16. Focus on success rate in terms of error probability for three different group sizes N ($N = 4$, $N = 6$ and $N = 8$ as expressed in each panel subtitle). Different curves show how the success rate varies for each error type - execution, program or delegate - shown in the figure legend on top. Other parameters used to reproduce the figure were: $\beta = 0.05$, $Z = 100$, risk probability $p = 0.9$, action space with $\#actions = 3$ and total number of rounds $r = 10$ following the parameters used in the figures of the main text. Two dotted-dashed lines reproduce the intersection points between program and execution errors as well as between delegate and execution errors as found in the main manuscript to facilitate comparison. Even though the intersection points between delegation and no-delegation might change for different N values, we always observe a region for positive but low error probability for which delegation achieves higher success than no-delegation both for program and for delegate error types.

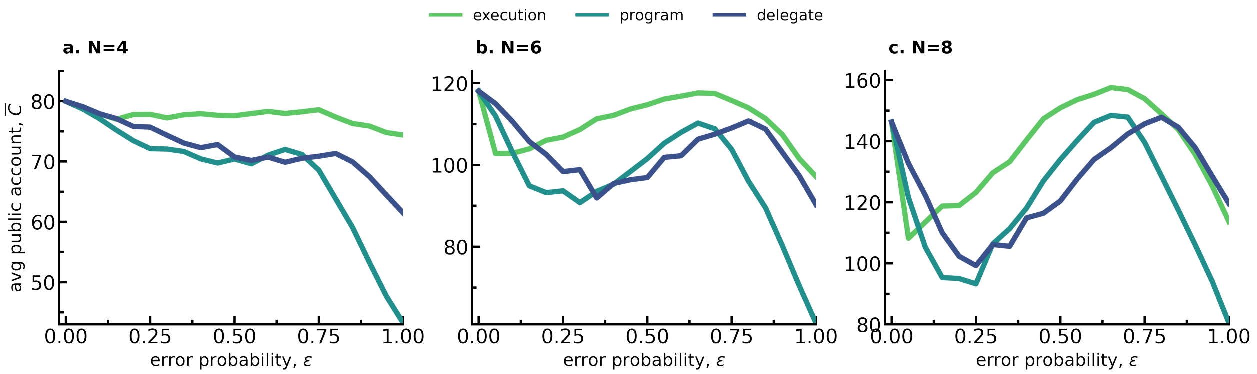

Supplementary Figure S17. Focus on average public account in terms of error probability for three different group sizes N ($N = 4$, $N = 6$ and $N = 8$ as expressed on each panel subtitle). Different curves show how the average public account values vary for each error type - execution, program or delegate - shown in the figure legend on top. Other parameters used to reproduce the figure were: $\beta = 0.05$, $Z = 100$, risk probability $p = 0.9$, action space with $\#actions = 3$ and total number of rounds $r = 10$ following the parameters used in the figures of the main text. Obviously the expected public account values change with group size since we have calculated the threshold M with regards to the group size N and initial endowment E: $M = NE/2$. Group size does not appear to affect the qualitative trends though: a sharp initial decrease in the public account values for execution error types also returns an initial region where delegation contributes more. However, this region is almost non-existent for $N = 4$, where the curve of the execution type is barely noticeable and so is its decrease in comparison with the delegation error types. Steepness of curves in all cases appear to increase with the increase of N.

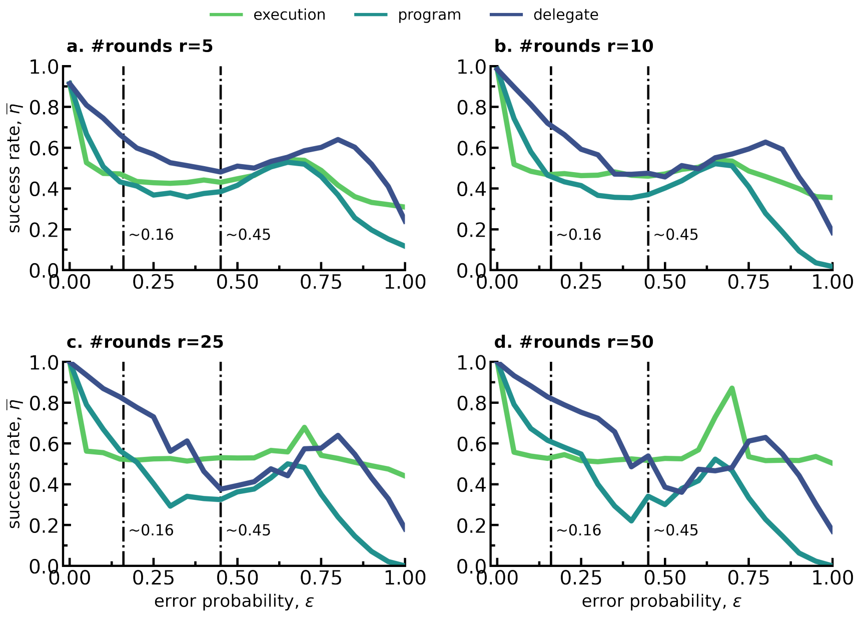

Supplementary Figure S18. Focus on success rate in terms of error probability for four different number of rounds r ($r = 5$, $r = 10$, $r = 25$ and $r = 50$ as expressed on each panel subtitle). Different curves show how the success rate varies for each error type - execution, program or delegate - shown in the figure legend on top. Other parameters used to reproduce the figure were: $\beta = 0.05$, $Z = 100$, risk probability $p = 0.9$, action space with $\#actions = 3$ and group size $N = 6$ following the parameters used in the figures of the main text. Two dotted-dashed lines reproduce the intersection points between program and execution errors as well as between delegate and execution errors as found in the main manuscript to facilitate comparison. Even though the specific points of intersection between delegation and no-delegation success rates might change, the general trends observed in the main manuscript remain robust with regards to a changing number of rounds.

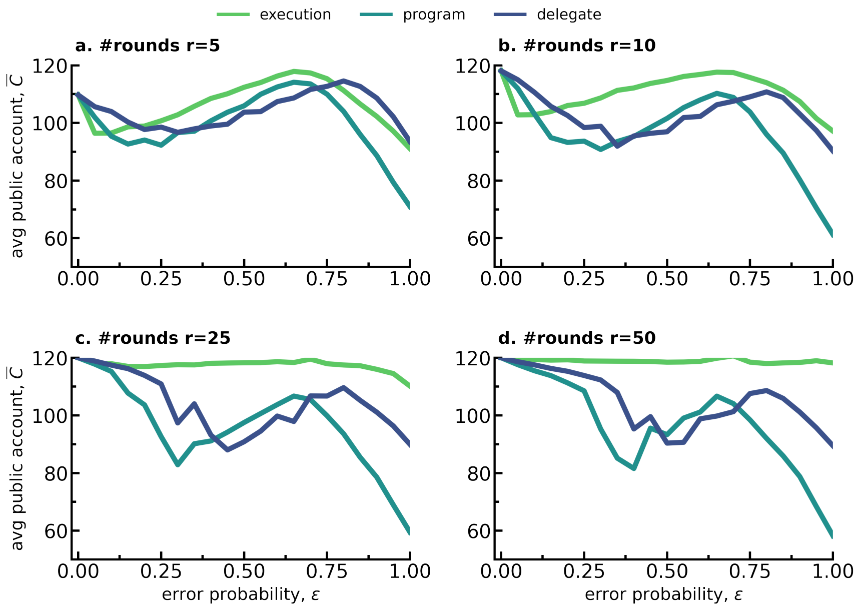

Supplementary Figure S19. Focus on average public account in terms of error probability for four different number of rounds r ($r = 5$, $r = 10$, $r = 25$ and $r = 50$ as expressed on each panel subtitle). Different curves show how the average public account values vary for each error type - execution, program or delegate - shown in the figure legend on top. Other parameters used to reproduce the figure were: $\beta = 0.05$, $Z = 100$, risk probability $p = 0.9$, action space with $\#actions = 3$ and group size $N = 6$ following the parameters used in the figures of the main text. With the increase of the number of rounds, the curve that represents execution errors becomes flatter, so that the intersection with the delegation curves appears for lower values of error probability or even disappears: when number of rounds is 50, execution errors always result in higher contributions to the public account.

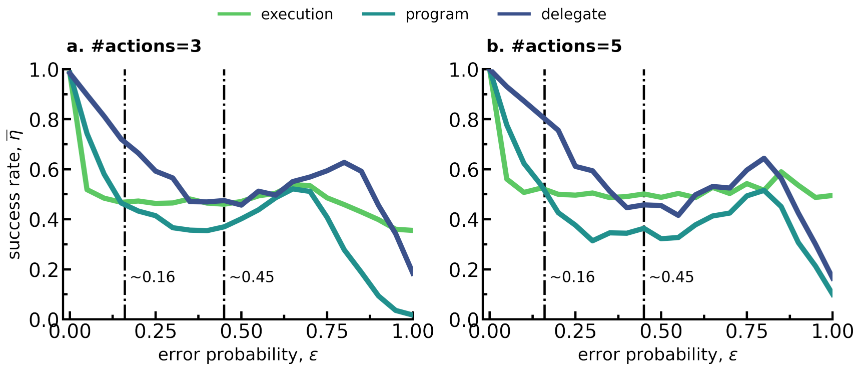

Supplementary Figure S20. Focus on success rate in terms of error probability for different action space granularity ($\#actions = 3$ and $\#actions = 5$ as expressed on each panel subtitle). Different curves show how the success rate varies for each error type - execution, program or delegate - shown in the figure legend on top. Other parameters used to reproduce the figure were: $\beta = 0.05$, $Z = 100$, risk probability $p = 0.9$, number of rounds $r = 10$ and group size $N = 6$ following the parameters used in the figures of the main text. Two dotted-dashed lines reproduce the intersection points between program and execution errors as well as between delegate and execution errors as found in the main manuscript to facilitate comparison. The granularity of the action space does not have a significant effect on the trends observed when comparing success between delegation and no-delegation mechanisms.

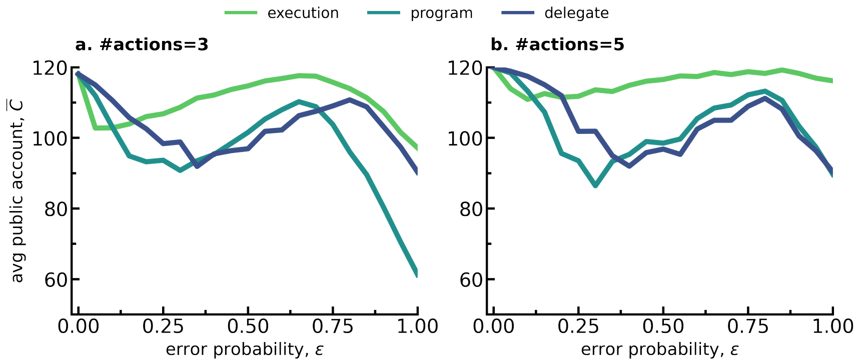

Supplementary Figure S21. Focus on average public account in terms of error probability for different action space granularity ($\#actions = 3$ and $\#actions = 5$ as expressed on each panel subtitle). Different curves show how the average public account values vary for each error type - execution, program or delegate - shown in the figure legend on top. Other parameters used to reproduce the figure were: $\beta = 0.05$, $Z = 100$, risk probability $p = 0.9$, number of rounds $r = 10$ and group size $N = 6$ following the parameters used in the figures of the main text. The granularity of the action space does not have a significant effect on the trends observed when comparing average public accounts between delegation and no-delegation mechanisms.

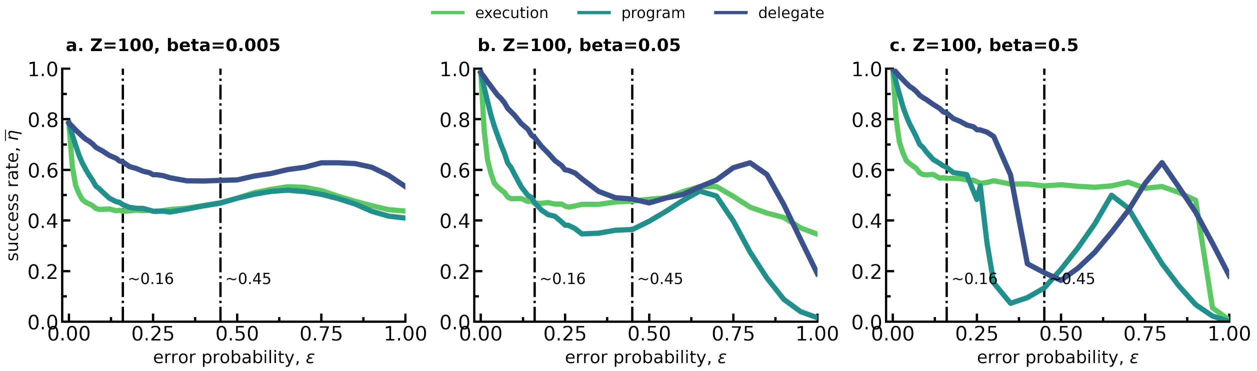

Supplementary Figure S22. Focus on success rate in terms of error probability for three different selection strengths β ($\beta = 0.005$, $\beta = 0.05$ and $\beta = 0.5$ as expressed on each panel subtitle). Different curves show how the success rate varies for each error type - execution, program or delegate - shown in the figure legend on top. Other parameters used to reproduce the figure were: $Z = 100$, risk probability $p = 0.9$, action space with $\#actions = 3$ and total number of rounds $r = 10$ following the parameters used in the figures of the main text. To estimate the payoffs, 10000 simulations were used instead of 1000 like in other figures of this SI. Two dotted-dashed lines reproduce the intersection points between program and execution errors as well as between delegate and execution errors as found in the main manuscript to facilitate comparison. Even though the specific points of intersection between delegation and no-delegation might change with β, the qualitative trends observed in the main manuscript remain unaltered: there is always a region for positive but low error probabilities for which the delegation populations exhibit higher success rates than the no-delegation one.

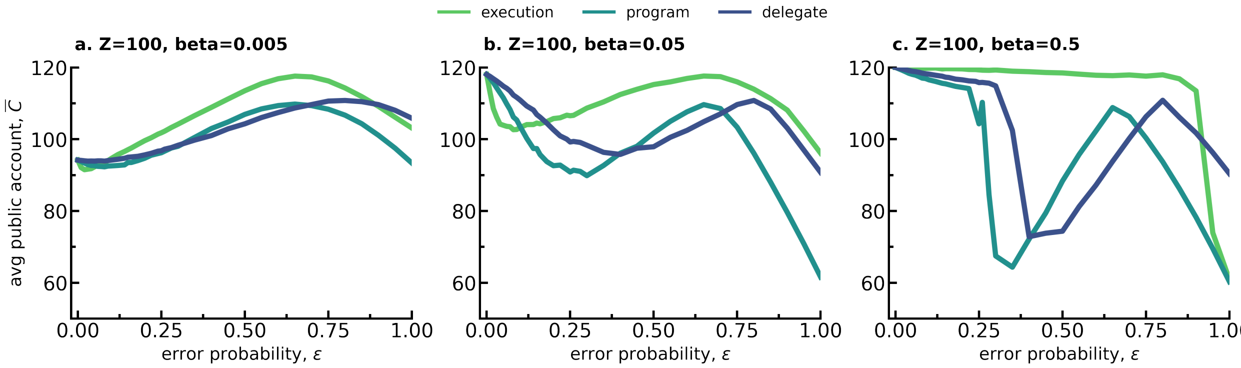

Supplementary Figure S23. Focus on average public account in terms of error probability for three different selection strengths β ($\beta = 0.005$, $\beta = 0.05$ and $\beta = 0.5$ as expressed on each panel subtitle). Different curves show how the average public account values vary for each error type - execution, program or delegate - shown in the figure legend on top. Other parameters used to reproduce the figure were: $Z = 100$, risk probability $p = 0.9$, action space with $\#actions = 3$ and total number of rounds $r = 10$ following the parameters used in the figures of the main text. To estimate the payoffs, 10000 simulations were used instead of 1000 like in other figures of this SI. Similarly to what happens when the number of rounds increase in Fig. 19, a higher selection strength might flatten the curve of the execution population so much that the intersection point disappears and this no-delegation population remains the highest contributor for the majority of the error probability range, unlike in the main manuscript.

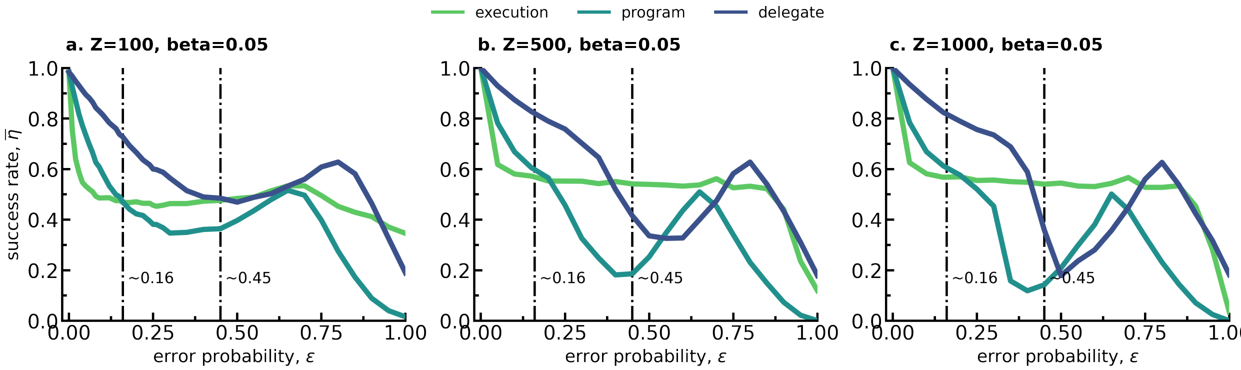

Supplementary Figure S24. Focus on success rate in terms of error probability for three different population size Z ($Z = 100$, $Z = 500$ and $Z = 1000$ as expressed on each panel subtitle). Different curves show how the success rate varies for each error type - execution, program or delegate - shown in the figure legend on top. Other parameters used to reproduce the figure were: $\beta = 0.05$, risk probability $p = 0.9$, action space with $\#actions = 3$ and total number of rounds $r = 10$ following the parameters used in the figures of the main text. To estimate the payoffs, 10000 simulations were used instead of 1000 like in other figures of this SI. Two dotted-dashed lines reproduce the intersection points between program and execution errors as well as between delegate and execution errors as found in the main manuscript to facilitate comparison. Increasing Z, similarly to β, results in steeper curves but does not affect the general trends observed already in the main manuscript when comparing delegation with no-delegation.

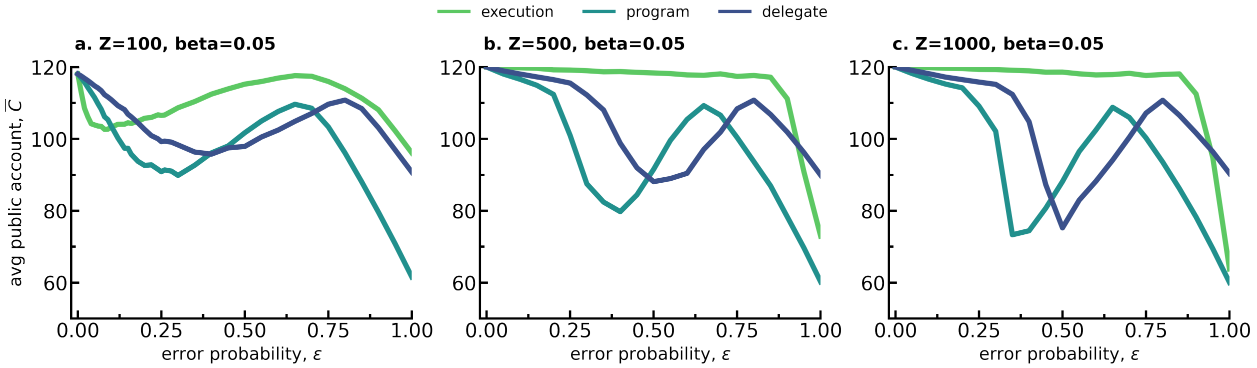

Supplementary Figure S25. Focus on average public account in terms of error probability for three different population size Z ($Z = 100$, $Z = 500$ and $Z = 1000$ as expressed on each panel subtitle). Different curves show how the average public account values vary for each error type - execution, program or delegate - shown in the figure legend on top. Other parameters used to reproduce the figure were: $\beta = 0.05$, risk probability $p = 0.9$, action space with $\#actions = 3$ and total number of rounds $r = 10$ following the parameters used in the figures of the main text. To estimate the payoffs, 10000 simulations were used instead of 1000 like in other figures of this SI. Increasing Z, similarly to β, results in the eventual disappearance of the intersection points between the no-delegation and the delegation curves, establishing the execution population as the highest contributor for the majority of the error probability range when Z is large.

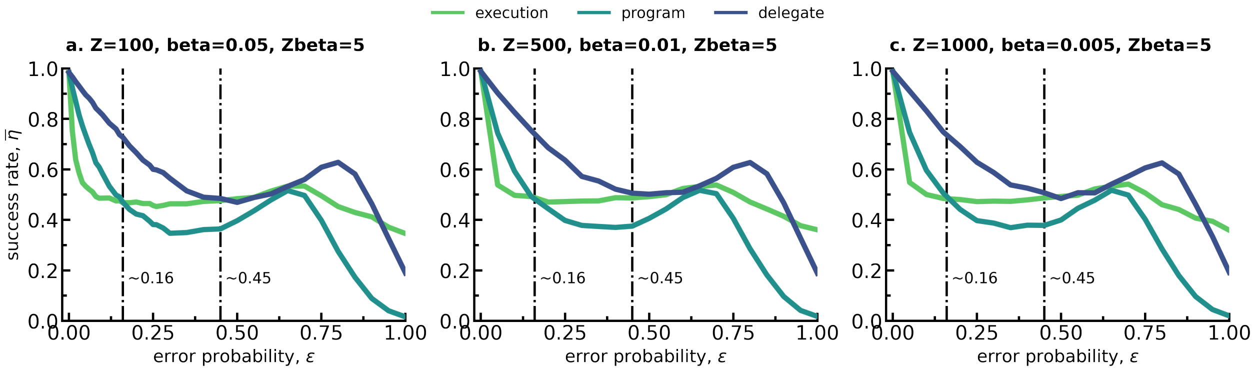

Supplementary Figure S26. Focus on success rate in terms of error probability for three different pairs of population size Z and selection strength β, however where $Z\beta$ is a constant equal to 5 (values expressed on each panel subtitle). Different curves show how the success rate varies for each error type - execution, program or delegate - shown in the figure legend on top. Other parameters used to reproduce the figure were: risk probability $p = 0.9$, action space with $\#actions = 3$ and total number of rounds $r = 10$ following the parameters used in the figures of the main text. To estimate the payoffs, 10000 simulations were used instead of 1000 like in other figures of this SI. Two dotted-dashed lines reproduce the intersection points between program and execution errors as well as between delegate and execution errors as found in the main manuscript to facilitate comparison. Maintaining $Z\beta$ constant, keeps the same function success rate in terms of error probability.

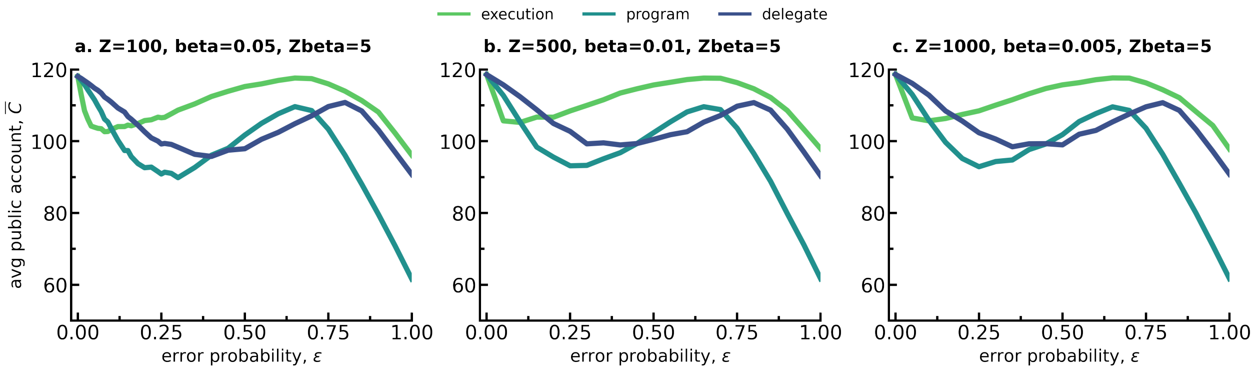

Supplementary Figure S27. Focus on average public account in terms of error probability for three different pairs of population size Z and selection strength β, however where $Z\beta$ is a constant equal to 5 (values expressed on each panel subtitle). Different curves show how the average public account values vary for each error type - execution, program or delegate - shown in the figure legend on top. Other parameters used to reproduce the figure were: risk probability $p = 0.9$, action space with $\#actions = 3$ and total number of rounds $r = 10$ following the parameters used in the figures of the main text. To estimate the payoffs, 10000 simulations were used instead of 1000 like in other figures of this SI. Maintaining $Z\beta$ constant, keeps the same function success rate in terms of error probability.

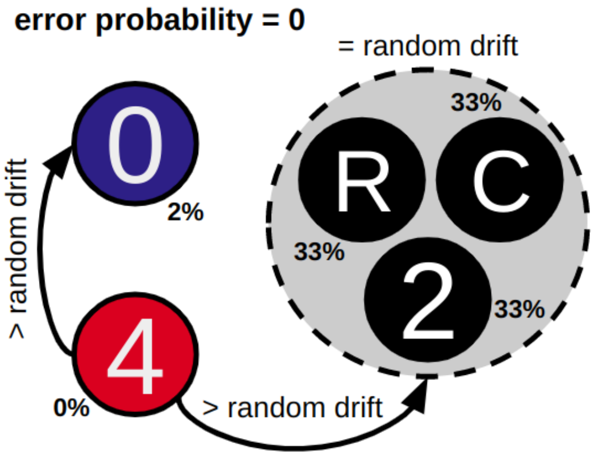

Supplementary Figure S28. Representation of the Markov chain that illustrates the transitions between the monomorphic states of the population when individuals commit no errors when implementing their strategies. Nodes *R*, *C*, *0*, *2* and *4* stand for the strategies Reciprocal, Compensatory, always-0, always-2 and always-4, respectively. The stationary distribution of each strategy within the evolutionary process is indicated with a number (rounded to the closest integer) and the % sign next to each node. Arrows represent transitions where a mutant from a strategy (arrow head) is able to invade a monomorphic population of another (arrow tail) with a fixation probability higher than random drift. A dashed circle around *R*, *C* and *2* represents the random drift like fixation that these strategies exhibit with one another. The values in the image are obtained for any of three previously mentioned error type populations (*execution*, *program* or *delegate*) but with error probability equal to 0. The other parameters used to reproduce this image are: $p = 0.9$, $r = 10$, $E = 40$, $A = \{0, 2, 4\}$, $N = 6$, $\beta = 0.05$, $Z = 100$, $\#sim = 1000$.

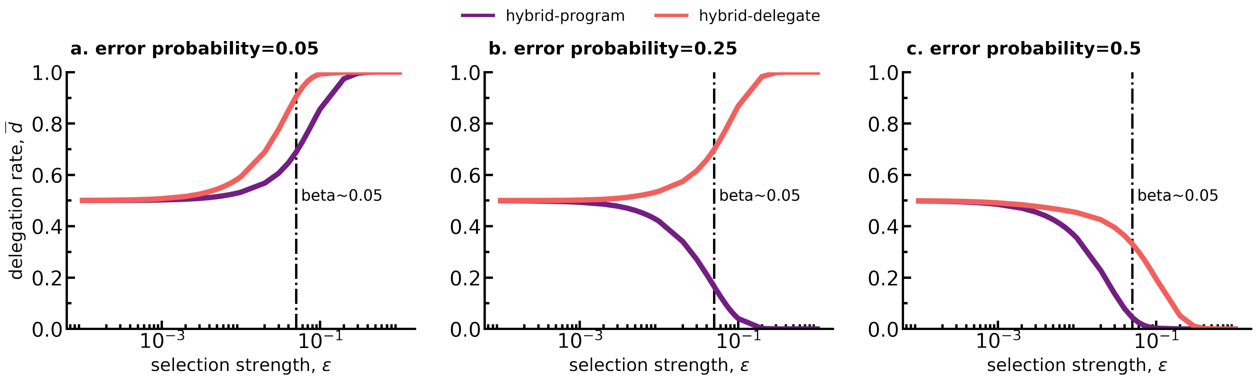

Supplementary Figure S29. Focus on delegation rate in terms of selection strength β for three different error probabilities ε ($\varepsilon = 0.05$, $\varepsilon = 0.25$ and $\varepsilon = 0.5$ as expressed on each panel subtitle). Different curves show how the success rate varies for each error type - execution, program or delegate - shown in the figure legend on top. Other parameters used to reproduce the figure were: $Z = 100$, risk probability $p = 0.9$, action space with $\#actions = 3$, total number of rounds $r = 10$ and group size $N = 6$, following the parameters used in the figures of the main text. To estimate the payoffs, 10000 simulations were used instead of 1000 like in other figures of this Supplementary Information. Moreover, we mark with a black dashed-dotted line the case portrayed in the main text - $\beta = 0.05$ - for reference. A logarithmic scale is used in the x-axis for selection strength, β.

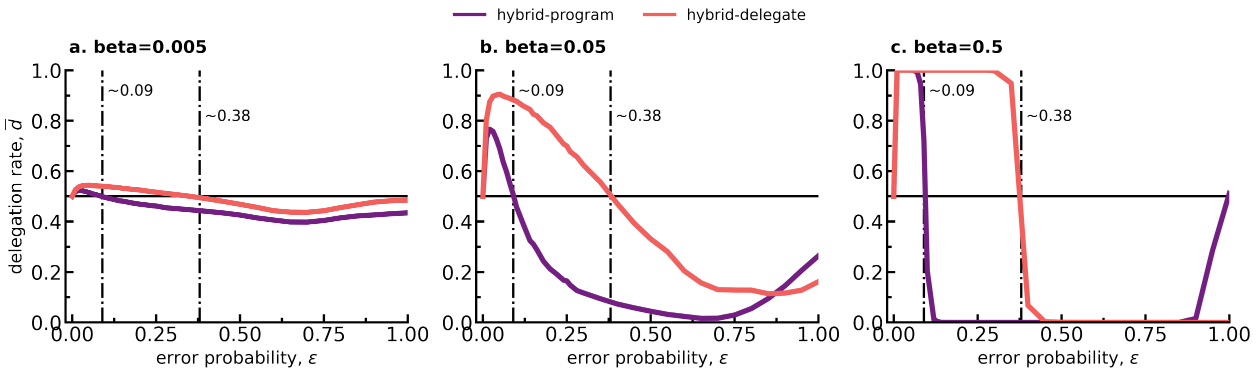

Supplementary Figure S30. Focus on delegation rate in terms of error probability for three different selection strengths β ($\beta = 0.005$, $\beta = 0.05$ and $\beta = 0.5$ as expressed on each panel subtitle). Different curves show how the success rate varies for each error type - execution, program or delegate - shown in the figure legend on top. Other parameters used to reproduce the figure were: $Z = 100$, risk probability $p = 0.9$, action space with $\#actions = 3$ and total number of rounds $r = 10$ following the parameters used in the figures of the main text. To estimate the payoffs, 10000 simulations were used instead of 1000 like in other figures of this Supplementary Information. Moreover, we mark with two black dashed-dotted lines when delegation rate drops below 50% for each delegation mechanism for the case portrayed in the main text - $\beta = 0.05$. As is known from literature, an increase in selection strength results in an increase of the steepness of the curves, but no change in the trends observed in the main manuscript are due to a variation in selection strength.

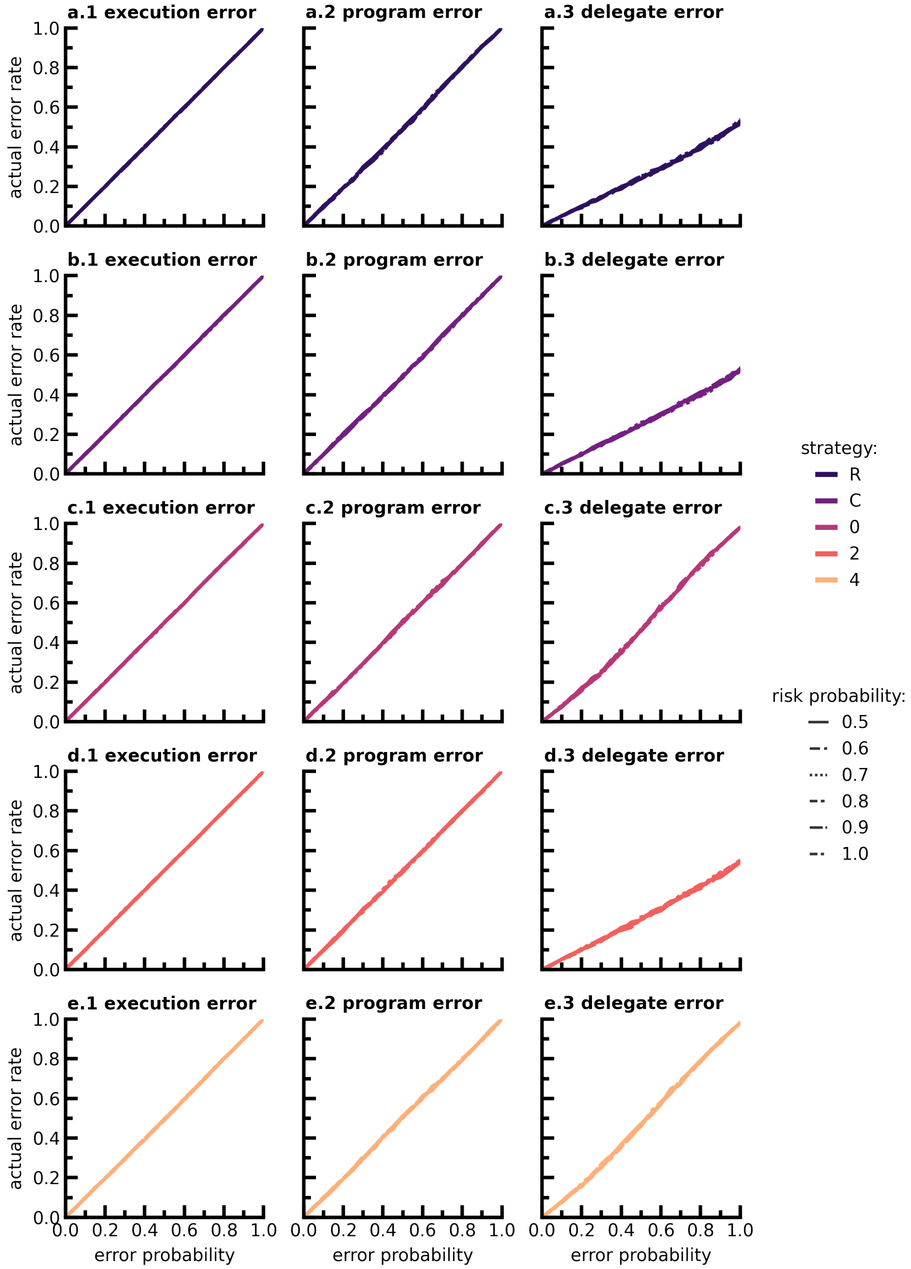

Supplementary Figure S31. Actual error rate vs error probability (ε) for all the three different error modes (execution error in column 1, program error in column 2 and delegate error in column 3), as well as for all the different monomorphic groups (following also the color scheme legend on the right), as represented by strategy: R(eciprocal) in row a, C(ompensatory) in row b, (always-)0 in row c, (always-)2 in row d, and (always-)4 in row e. Moreover, for each panel we show how the function would change for different risk probabilities according to the style legend on the right where the following risk probabilities are represented: 0.5, 0.6, 0.7, 0.8, 0.9 and 1. By analyzing the figure, we can see that in general the actual error rates varies (close to) linearly to the error probability and varies little with different risk probabilities, strategies or error modes. The only cases where a great difference is observed is for the case of strategies R(eciprocal), C(ompensatory) and (always-)2 only in the delegate error case (see panels a.3, b.3 and d.3). In this manner, we believe that our choice to work with both program and delegate errors to analyze delegation (in comparison with no-delegation) is justified, by using both a delegation method where the actual error rate is approximated to the one exhibited by non-delegated action and another where it is expected to be very different.
